# Health effects associated with vegetable consumption: a Burden of Proof study

**DOI:** 10.1038/s41591-022-01970-5

**Published:** 2022-10-10

**Authors:** Jeffrey D. Stanaway, Ashkan Afshin, Charlie Ashbaugh, Catherine Bisignano, Michael Brauer, Giannina Ferrara, Vanessa Garcia, Demewoz Haile, Simon I. Hay, Jiawei He, Vincent Iannucci, Haley Lescinsky, Erin C. Mullany, Marie C. Parent, Audrey L. Serfes, Reed J. D. Sorensen, Aleksandr Y. Aravkin, Peng Zheng, Christopher J. L. Murray

**Affiliations:** 1 Institute for Health Metrics and Evaluation, University of Washington, Seattle, WA USA; 2 Department of Health Metrics Sciences, School of Medicine, University of Washington, Seattle, WA USA; 3 University of British Columbia, Vancouver, British Columbia Canada; 4 Department of Applied Mathematics, University of Washington, Seattle, WA USA

**Keywords:** Risk factors, Cardiovascular diseases, Cancer

## Abstract

Previous research suggests a protective effect of vegetable consumption against chronic disease, but the quality of evidence underlying those findings remains uncertain. We applied a Bayesian meta-regression tool to estimate the mean risk function and quantify the quality of evidence for associations between vegetable consumption and ischemic heart disease (IHD), ischemic stroke, hemorrhagic stroke, type 2 diabetes and esophageal cancer. Increasing from no vegetable consumption to the theoretical minimum risk exposure level (306–372 g daily) was associated with a 23.2% decline (95% uncertainty interval, including between-study heterogeneity: 16.4–29.4) in ischemic stroke risk; a 22.9% (13.6–31.3) decline in IHD risk; a 15.9% (1.7–28.1) decline in hemorrhagic stroke risk; a 28.5% (−0.02–51.4) decline in esophageal cancer risk; and a 26.1% (−3.6–48.3) decline in type 2 diabetes risk. We found statistically significant protective effects of vegetable consumption for ischemic stroke (three stars), IHD (two stars), hemorrhagic stroke (two stars) and esophageal cancer (two stars). Including between-study heterogeneity, we did not detect a significant association with type 2 diabetes, corresponding to a one-star rating. Although current evidence supports increased efforts and policies to promote vegetable consumption, remaining uncertainties suggest the need for continued research.

## Abstract (web summary)

A meta-analysis using the Burden of Proof function identified modest evidence supporting a protective role of vegetable consumption against ischemic heart disease, stroke and esophageal cancer but not type 2 diabetes.

## Main

Previous research suggests that greater levels of vegetable consumption are associated with lower risk of several adverse health outcomes, including ischemic heart disease (IHD)^1–3^, stroke^4^, type 2 diabetes^5^ and esophageal cancer^6^. The 2019 Global Burden of Diseases, Injuries, and Risk Factors Study (GBD) estimated that 429,000 (95% uncertainty interval (UI) = 340,000–718,000) deaths and 13.0 million (8.25–17.5) disability-adjusted life years (DALYs) were attributable to low vegetable consumption globally in 2019 (ref. ^7^). The large estimated burden demonstrates the importance of more fully understanding the relationship between vegetable consumption and possible health outcomes and the strength of evidence supporting the understanding of those relationships.

Prior meta-analyses have estimated an 18% (95% confidence interval (CI): 8–17) reduction in IHD risk when comparing 0–400 g of vegetables daily^1^; a 16% (95% CI: 10–21) reduction in IHD risk when comparing 0–200 g daily^2^; and an 8% (95% CI: 4–13) lower incidence of IHD and a 14% (95% CI: 11–17) lower IHD mortality between individuals in the highest versus lowest quantiles of vegetable consumption^3^. Similar reductions have been estimated for ischemic stroke risk, with Hu et al.^4^ estimating a relative risk (RR) of 0.86 (95% CI: 0.79–0.93) when comparing each study’s highest and lowest categories of vegetable consumption. Regarding vegetable consumption and type 2 diabetes, previous meta-analyses have offered mixed results. Whereas Schwingshackl et al.^5^ suggested a statistically significant RR of 0.92 (95% CI: 0.89–0.95) when comparing consumption of 240 g per day versus 0 g per day, Halvorsen et al.^8^ did not detect a significant association when comparing type 2 diabetes risk between those consuming 200 g per day and 0 g per day (RR: 0.97, 95% CI: 0.94–1.01)^8^. Similarly, the results of meta-analyses of vegetable consumption and esophageal squamous cell carcinoma (ESCC) have also been mixed. Whereas Liu et al.^6^ found significantly lower ESCC risk associated with increased vegetable consumption (RR = 0.56, 95% CI: 0.45–0.69 comparing highest to lowest vegetable consumption groups)^6^, Vingeliene et al.^9^ did not detect a significant relationship (RR = 0.98, 95% CI: 0.90–1.06 comparing 100 g to 0 g of vegetables daily)^9^. Other meta-analyses have found significant associations between vegetable consumption and esophageal adenocarcinoma risk, with an RR of 0.89 (95% CI: 0.80–0.99) for 100 g per day of vegetable consumption versus 0 g per day^9^ and an RR of 0.76 (95% CI: 0.59–0.96) for the highest versus lowest vegetable consumption groups^10^.

An understanding of the direction and magnitude of associations between vegetable consumption and adverse health outcomes, as well as the strength of the evidence supporting the existence of these associations, is essential for guiding policy and individual health decisions related to diet and agriculture. Although many of the meta-analyses provide 95% CIs on their dose–response curves, this statistical uncertainty does not capture uncertainty that might stem from differences in study design (for example, experimental versus observational designs and completeness of confounder adjustment), potential publication bias, between-study heterogeneity or small numbers of studies. Zurbau et al.^11^ addressed the question of evidence quality and applied the Grading of Recommendations Assessment, Development and Evaluation (GRADE) framework to provide a qualitative summary rating of the evidence. They rated the quality of the evidence for the relationship between vegetable consumption and IHD mortality risk as ‘moderate’ and the evidence for the relationship between vegetable consumption and IHD incidence risk as ‘low’^3^. The GRADE framework takes into account bias in the data, imprecision in estimation for a particular study, inconsistency among studies, indirectness of the research question of the included study to the research question of study at hand and publication bias^11^. However, the framework is both qualitative and subjective and, thus, has limited utility for comparing the strength of evidence between risk factors being evaluated by different groups of researchers. Most recently, Schwingshackl et al.^12^ developed NutriGrade, a nutrition-specific scoring system to evaluate quality of evidence tailored to deal with the particular challenges of nutrition studies that includes assessment of risk of bias, study quality, study limitations, precision, heterogeneity, directness, publication bias, funding bias, study design, effect size and dose response. NutriGrade is a promising approach but uses a scoring system that assigns points with limited empirical basis, and, with its orientation toward nutrition research, it may not be readily applicable or appropriate for evaluating the larger collection of risk factors included in the GBD study. The Hierarchies of Evidence Applied to Lifestyle Medicine (HEALM) was developed as a construct to evaluate the strength of evidence for social, behavioral and genetic risk factors for which randomized trials are impossible, impractical or unethical^13^. Although offering a useful construct for considering evidence beyond trials, HEALM relies largely on subjective expert assessments and is not applicable or appropriate for all risk factors included in the GBD study. The Newcastle-Ottawa scale^4,8,10^ provides a framework for assessing the risk of bias of observational studies with regard to participant selection and comparability and the ascertainment of exposures and outcomes. This framework is useful, but, similarly to the GRADE framework, it faces limitations as a subjective and qualitative assessment with poor inter-rater reliability and does not offer a mechanism to synthesize the quality and strength of evidence across all relevant studies to generate a global assessment^14^.

The standard meta-analytic approach of reporting mean effects offers an objective and quantitative measure of the strength of associations observed in epidemiological studies, but it offers no assessment of the extent to which we are to believe that the mean effect accurately represents the true association versus a manifestation of biases and errors. Conversely, existing methods to judge the quality of the evidence (for example, GRADE) offer a means to assess the quality of the underlying evidence, but they do so subjectively and do not extend to estimating what the true effect size might be in light of any biases or other limitations of the evidence.

As part of the GBD study, Zheng et al.^15^ developed a Bayesian meta-regression tool and methods for assessing the quality of evidence for risk–outcome associations that are both objective and quantitative^15^. This approach is intended to complement and bridge the gap between quantitative meta-analyses and subjective assessments of evidentiary strength. It offers a means to evaluate the strength of epidemiological evidence for associations between risk–outcome pairs using an objective and empirical approach within a transparent regression framework and connects this to the estimate of the mean effect by reporting the strength of the association that is consistent with the most conservative interpretation of the evidence. A burden of proof risk function (BPRF) can then be calculated from this conservative risk function as the 5th (for harmful) or 95th quantile risk curve that is closest to the null, and this BRPF represents the weakest association across the range of exposures that is consistent with the available data: the farther the BRPF is from the null, the stronger the evidence is for that association^15,16^. Finally, the exposure-averaged BPRF for a risk–outcome pair is converted into a risk–outcome score (ROS) that can be compared across risk–outcome pairs and assigned a star rating (from one to five) based on the quantitative assessment of the association^15^.

For this study, we applied this meta-regression approach to both quantify health effects of vegetable consumption and evaluate the quality of the evidence supporting those effects. We conducted a systematic review to identify studies that estimated associations between vegetable consumption and the five health outcomes for which we estimate burden attributable to low vegetable consumption in the GBD study: type 2 diabetes, esophageal cancer, hemorrhagic stroke, IHD and ischemic stroke. These outcomes were selected across previous iterations of the GBD study as those for which there existed convincing or probable evidence for a causal effect based on the World Cancer Research Fund Grading System^17^ and for which there existed sufficient data to enable estimation of all components of the modeling chain. The main findings and policy implications of the current study are summarized in Table 1.

**Table 1:** Policy summary

Background	Characterizing associations between vegetable consumption and adverse health outcomes, as well as the strength of the evidence supporting the existence of these associations, is essential for guiding relevant policy and individual health decisions. Although high-quality meta-analyses have previously assessed the effects of vegetable consumption, none has formally assessed evidence strength. We applied a meta-regression tool to estimate the mean risk function and quantify the quality of evidence for the association between vegetable consumption and five health outcomes: IHD, ischemic stroke, hemorrhagic stroke, type 2 diabetes and esophageal cancer.
Main findings and limitations	We estimated that the TMREL for vegetable consumption is between 306 g and 372 g daily. Increasing vegetable consumption from 0 g per day to the theoretical minimum risk exposure level (306–372 g daily) was associated with a 23.2% decline (95% UI, inclusive of between-study heterogeneity: 16.4–29.4) in ischemic stroke mean risk; a 22.9% (13.6–31.3) decline in mean risk of IHD; a 15.9% (1.7–28.1) decline in hemorrhagic stroke mean risk; a 28.5% (−0.02 to 51.4) decline in esophageal cancer mean risk; and a 26.1% (−3.6 to 48.3) decline in type 2 diabetes mean risk. We found statistically significant evidence of publication bias with IHD and esophageal cancer, and evidence of study-level biases for IHD and type 2 diabetes. We found statistically significant evidence of a protective effect of vegetable consumption for ischemic stroke, IHD, hemorrhagic stroke and esophageal cancer, with ROSs of 0.15, 0.13, 0.02 and 0.001, respectively, corresponding to a three-star rating for ischemic stroke and two-star ratings for the latter three outcomes. Based on a conservative interpretation of the evidence that accounted for between-study heterogeneity, we did not detect statistically significant associations between vegetables and type 2 diabetes, leading to this pair being classified as one-star. The ROSs for ischemic stroke and IHD reflect the reasonably good quality and number of studies, large sample sizes and little between-study heterogeneity on the one hand and modest magnitude of the effect sizes on the other. Although the evidence remains compelling for hemorrhagic stroke and esophageal cancer, it is weakened by modest effect sizes, relatively limited number of studies and large between-study heterogeneity. The quality of our estimates and the strength of the evidence for the health effects of vegetable consumption are limited by the strong potential for confounding in estimates of associations between diet and health and the potential for exposure misclassification in particular. We identified no relevant randomized trials, and, because vegetable consumption is strongly associated with other aspects of diet and other demographic, social and behavioral risk factors (for example, age, income, education, physical activity and smoking), some degree of residual confounding likely remains.
Policy implications	RCTs would help to definitively clarify the extent to which residual confounding and bias may distort estimates from observational studies, and, to the extent that they confirm the findings of observational studies, they should improve confidence in these associations. Still, we found sufficient evidence to justify more robust efforts and policies to promote increased vegetable consumption to reduce chronic disease risk, especially with regard to IHD, stroke and esophageal cancer. Such efforts must address the availability, affordability and acceptability of vegetables, including efforts focused on the food system to incentivize increased vegetable production and reduce waste; efforts to increase affordability through agricultural or consumer subsidies; and efforts to increase acceptability and demand for vegetables through increased public awareness of the health benefits of vegetable consumption, advertising and labeling to empower consumer choice and making vegetable-rich foods more broadly available in restaurants and institutional settings.

## Results

### Overview

**Extended Data Fig. 1:**
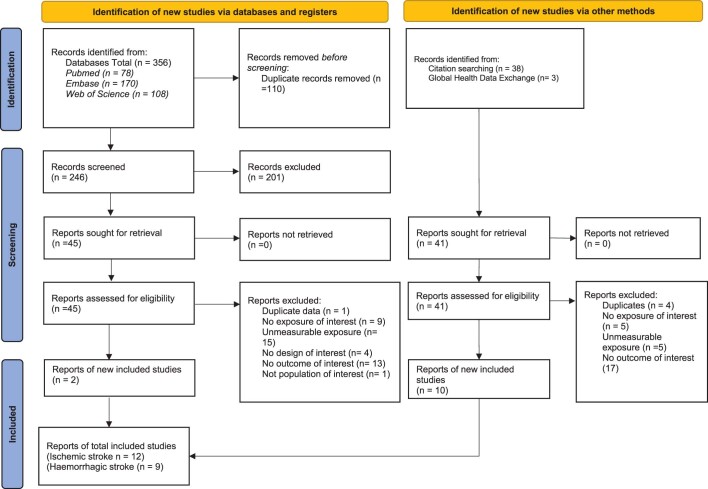
PRISMA flow diagram of vegetable consumption and stroke subtypes (ischemic stroke and hemorrhagic stroke). The PRISMA flow diagram covering the data seeking approach for vegetable consumption and stroke subtypes (ischemic stroke and hemorrhagic stroke). Template is from: Page MJ, McKenzie JE, Bossuyt PM, Boutron I, Hoffmann TC, Mulrow CD, et al. The PRISMA 2020 statement: an updated guideline for reporting systematic reviews. BMJ 2021;372:n71. doi: 10.1136/bmj.n71. For more information, visit: http://www.prisma-statement.org/.

**Extended Data Fig. 2:**
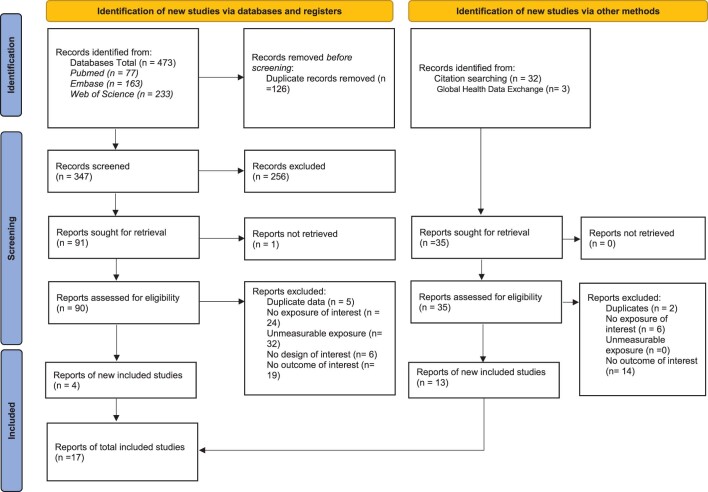
PRISMA flow diagram of vegetable consumption and IHD. The PRISMA flow diagram covering the data seeking approach for vegetable consumption and IHD. Template is from: Page MJ, McKenzie JE, Bossuyt PM, Boutron I, Hoffmann TC, Mulrow CD, et al. The PRISMA 2020 statement: an updated guideline for reporting systematic reviews. BMJ 2021;372:n71. doi: 10.1136/bmj.n71. For more information, visit: http://www.prisma-statement.org/.

**Extended Data Fig. 3:**
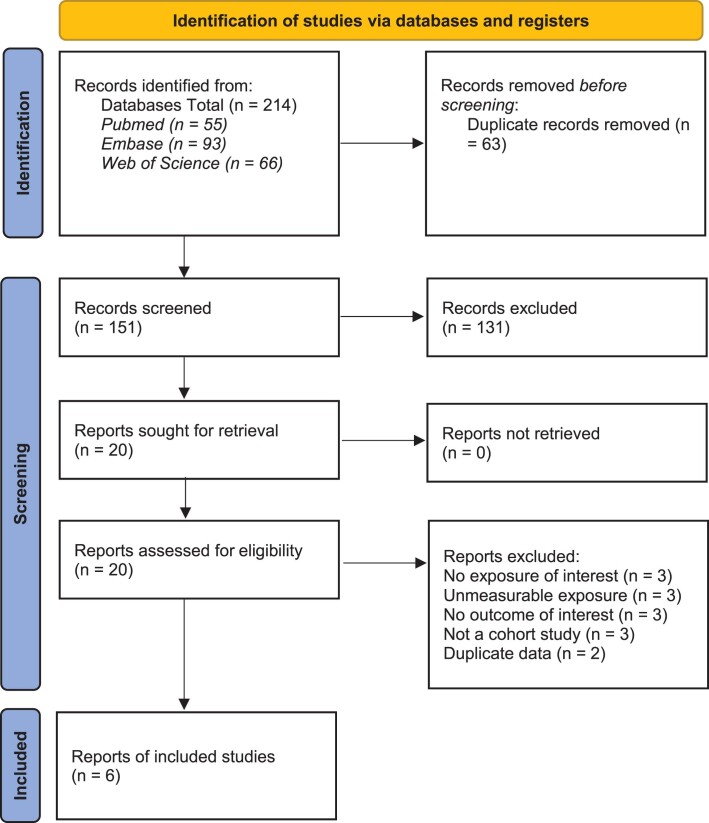
PRISMA flow diagram of vegetable consumption and esophageal cancer. The PRISMA flow diagram covering the data seeking approach for vegetable consumption and esophageal cancer. Template is from: Page MJ, McKenzie JE, Bossuyt PM, Boutron I, Hoffmann TC, Mulrow CD, et al. The PRISMA 2020 statement: an updated guideline for reporting systematic reviews. BMJ 2021;372:n71. doi: 10.1136/bmj.n71. For more information, visit: http://www.prisma-statement.org/.

**Extended Data Fig. 4:**
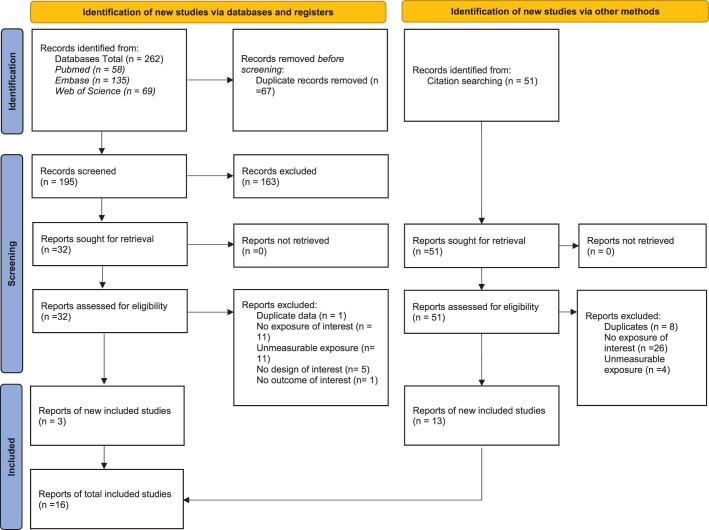
PRISMA flow diagram of vegetable consumption and type 2 diabetes. The PRISMA flow diagram covering the data seeking approach for vegetable consumption and type 2 diabetes. Template is from: Page MJ, McKenzie JE, Bossuyt PM, Boutron I, Hoffmann TC, Mulrow CD, et al. The PRISMA 2020 statement: an updated guideline for reporting systematic reviews. BMJ 2021;372:n71. doi: 10.1136/bmj.n71. For more information, visit: http://www.prisma-statement.org/.

Following the Preferred Reporting Items for Systematic Reviews and Meta-Analyses (PRISMA) guidelines^18^, we systematically searched the literature for studies reporting associations between vegetable consumption and each of the five included health outcomes, and we screened records based on pre-defined inclusion and exclusion criteria (Methods). The review workflow is detailed for each health outcome in the PRISMA flow diagrams (Extended Data Figs. 1–4 and Supplementary Tables 1 and 2). We identified 50 eligible studies^19–68^ presenting a total of 247 estimates of RRs for associations between vegetable consumption and the five included health outcomes. All included studies except one were of prospective cohort design. Forty-four studies used food frequency questionnaires to assess vegetable consumption^19–62^; three studies used dietary recall^63–65^; one study used 7-day food record^66^; one study used a 7-day diet history^67^; and one study used a self-administered questionnaire^68^. Individual vegetable consumption levels across studies ranged from 0 g to 1,177 g per day. Follow-up periods ranged from 3 years to 24 years, with a median follow-up of 11 years (interquartile range = 8.0–15.0). Data collection across studies occurred between 1967 and 2020 and included 4,609,296 participants from 34 countries and territories (Supplementary Table 3).

### Minimum risk level of vegetable intake

Increased vegetable consumption was associated with reduced risk of all included outcomes, and we subsequently selected the theoretical minimum risk exposure level (TMREL) to correspond to high real-world consumption levels for which we had adequate data to draw robust conclusions about risk. This TMREL was based on observed exposure levels reported in the included studies (see Methods for details), in which the midpoint of the high consumption category ranged from 16 g per day to 712 g per day across studies, with a mean of 229 g per day, and the upper bound ranged from 22 g per day to 1,177 g per day. We estimated a TMREL of 306–372 g per day, corresponding to approximately 4–5 servings per day.

### Vegetable consumption and ischemic stroke

We identified 12 prospective cohort studies^19–29,68^ to evaluate the relationship between vegetable consumption and ischemic stroke among 829,610 individuals, with a median follow-up of 13.2 years (follow-up range: 3.1–24 years). Six cohorts were from Europe^22–27^; four cohorts were from Asia^19–21,68^; and two cohorts were from North America^28,29^. Ten studies determined outcomes using administrative medical records or disease registries^20–29^; one study used self-reported incidence^19^; and one study used death certificates^68^. In all studies, the effect size measure was adjusted for body mass index (BMI), whereas eight studies adjusted for type 2 diabetes^20,21,23–27,68^; 11 studies adjusted for physical activity^19–29^; six studies adjusted for hypertension^20,21,26–28,68^; and ten studies adjusted for alcohol intake^19,21,23–25,27–29,68^. Seven studies reported effect sizes with hazard ratios (HRs)^19–21,23,27,29,68^, and the rest reported RRs^22,24–26,28^.

**Fig. 1:**
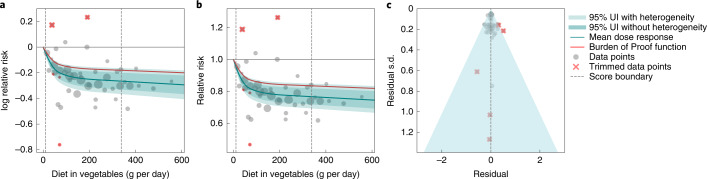
Vegetable consumption and ischemic stroke. **a**, log RR. **b**, RR function. **c**, A modified funnel plot showing the residuals (relative to zero) on the *x* axis and the estimated s.d. that includes reported s.d. and between-study heterogeneity on the *y* axis.

We observed a statistically significant, non-linear, monotonic decline in ischemic stroke risk associated with higher vegetable consumption (Fig. 1). The largest difference in risk was observed when comparing risk between zero and low consumption levels, with more modest marginal reductions in ischemic stroke risk observed with comparisons between moderate and high consumption. The mean risk of ischemic stroke at 100 g per day was 19.8% (13.9–25.1 including between-study heterogeneity) lower than at 0 g per day, whereas it was just 2.8% (1.9–3.7) lower at 200 g per day compared to 100 g per day; 1.1% (0.8–1.5) lower at 300 g per day compared to 200 g per day; and 0.7% (0.5–1.0) lower at 400 g per day compared to 300 g per day. Compared to the TMREL (306–372 g per day), consuming no vegetables was associated with a 23.2% (16.4–29.4) greater mean risk of ischemic stroke.

A conservative (closest to null) interpretation of the evidence suggests that the average vegetable intake (that is, levels between the 15th and 85th percentiles) was associated with a 14.2% lower risk of ischemic stroke compared to 0 g of vegetable intake. We converted this BPRF to an ROS of 0.15, corresponding to a three-star rating. Ratings of two through five translate to associations that remain statistically significant after accounting for between-study heterogeneity, with a three-star rating indicating that average exposure decreases risk by 13–34% compared to no exposure. Our analysis did not find significant evidence of publication bias, and visual inspection of the funnel plot provides no evidence of substantial publication bias (Fig. 1c).

### Vegetable consumption and IHD

Our analysis of vegetable consumption and IHD included 78 observations from 17 prospective cohort studies^21,32,34,37,39,40,42,46,49,50,53,54,57,60,61,66,67^, including a total of 1,952,226 participants and 41,494 IHD events. Five studies used IHD incidence^32,40,46,60,61^ as the endpoint to estimate effect sizes; four studies used mortality^39,53,54,57^; and eight studies used both IHD incidence and mortality^21,34,37,42,49,50,66,67^. Fourteen studies determined outcomes using administrative medical records, disease registries or death certificates^21,32,34,37,39,40,49,50,53,54,57,61,66,67^; one study supplemented records with self-reported incidence^42^; and two studies used physician diagnosis^46,60^. All studies but one adjusted their effect size measure for age^21,32,34,37,39,40,42,46,49,50,53,54,57,60,61,67^, and ten studies either adjusted for or stratified by sex^21,37,40,46,49,57,60,61,66,67^. Other common adjustment variables include smoking (*n* = 16)^23–27,29–31,69^, alcohol consumption (*n* = 14)^21,32,34,37,40,42,49,50,53,54,57,60,61,67^, BMI (*n* = 15)^21,32,34,37,39,40,42,49,50,53,54,60,61,66,67^, physical activity (*n* = 15)^32,34,37,39,42,46,49,50,53,54,57,60,61,66,67^, education level (*n* = 11)^34,37,46,49,50,53,54,57,60,66,67^, hypertension (*n* = 9)^21,34,37,39,42,49,53,54,60^ and type 2 diabetes (*n* = 8)^21,34,37,42,46,49,54,60^. Twelve studies reported effect sizes with HRs^21,37,40,46,49,53,54,57,60,61,66,67^, and five reported RRs^32,34,39,42,50^.

**Fig. 2:**
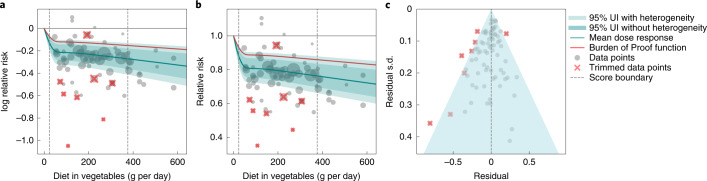
Vegetable consumption and IHD. **a**, log RR function. **b**, RR function. **c**, A modified funnel plot showing the residuals (relative to zero) on the *x* axis and the estimated s.d. that includes reported s.d. and between-study heterogeneity on the *y* axis.

We observed a statistically significant, non-linear monotonic decline in IHD risk associated with higher vegetable consumption (Fig. 2). The largest difference in risk was observed when comparing risk between zero and low consumption levels, with more modest marginal reductions in IHD risk observed with comparisons between moderate and high consumption. The mean risk of IHD at 100 g per day was 19.3% (95% UI including between-study heterogeneity = 11.3–26.3) lower than at 0 g per day, whereas it was just 1.5% (0.8–2.1) lower at 200 g per day compared to 100 g per day; 2.2% (1.2–3.1) lower at 300 g per day compared to 200 g per day; and 1.7% (1.0–2.4) lower at 400 g per day compared to 300 g per day.

Compared to the TMREL (306–372 g per day), consuming no vegetables was associated with a 22.9% (13.6–31.3, inclusive of between-study heterogeneity) greater mean risk of IHD. Notably, the uncertainty around the risk curve is modest, and we observed little difference in uncertainty whether the effect of between-study heterogeneity was included or not (Fig. 2).

Using the BPRF analysis, we found that average vegetable consumption levels (those falling between the 15th and 85th percentiles of exposure observed in the included studies) were associated with a 12.1% decreased risk of IHD compared to 0 g of consumption. This is a conservative estimate that corresponds to the smallest reduction in risk (closest to null) that is consistent with the available evidence and inclusive of between-study heterogeneity and other forms of uncertainty. This BPRF equated to an ROS of 0.13, corresponding to a two-star rating. Ratings of two through five translate to associations that remain statistically significant after accounting for between-study heterogeneity, with a two-star rating indicating that average exposure decreases risk by 0–13% compared to no exposure. We found that trimming had little effect on the ROS, and, without trimming, the score increased to 0.127, suggesting that the ROS for vegetable consumption and IHD is relatively insensitive to outliers. Although we observed little asymmetry in the funnel plot (Fig. 2c), the result of the Egger’s regression suggested statistically significant evidence of small-study bias (Egger’s regression *P* *=* 0.044).

### Vegetable consumption and hemorrhagic stroke

Our analysis of vegetable consumption and hemorrhagic stroke included 30 observations from nine prospective cohort studies^19–23,25–27,68^, including a total of 646,338 participants. Eight studies determined outcomes using administrative medical records^20–23,25–27,68^, disease registries or death certificates, and one study used self-reported incidence^19^. All studies adjusted for age; six studies adjusted for other components of diet^20,21,23,26,27,68^; and eight studies either adjusted for or stratified by sex^19–23,26,27,68^. Other common adjustment variables included smoking (*n* = 9)^23–27,29–31,69^, alcohol consumption (*n* = 7)^19,21,23,25–27,68^, BMI (*n* = 9)^23–27,29–31,69^, physical activity (*n* = 8)^19–23,25–27^, education level (*n* = 4)^20,23,26,27^, hypertension (*n* = 5)^20,21,26,27,68^ and type 2 diabetes (*n* = 7)^20,21,23,25–27,68^.

**Fig. 3:**
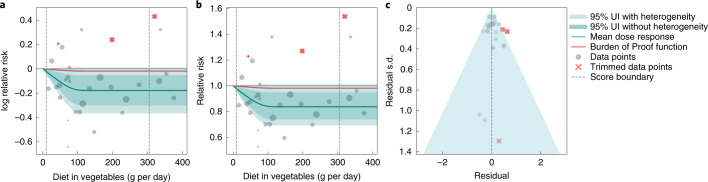
Vegetable consumption and hemorrhagic stroke. **a**, log RR function. **b**, RR function. **c**, A modified funnel plot showing the residuals (relative to zero) on the *x* axis and the estimated s.d. that includes reported s.d. and between-study heterogeneity on the *y* axis.

We observed a statistically significant, non-linear, monotonic decline in hemorrhagic stroke risk with increasing vegetable consumption (Fig. 3). The largest difference in risk was observed when comparing risk between 0 g per day and 100 g per day, with little additional marginal risk reduction observed with consumption levels greater than 100 g per day. The mean risk of hemorrhagic stroke at 100 g per day was 16.0% lower (95% UI including between-study heterogeneity = 1.6–27.7) than at 0 g per day, whereas it was just 0.3% (0.03–0.6) lower at 200 g per day compared to 100 g per day.

Compared to the TMREL, consuming no vegetables was associated with a 15.9% (1.7–28.1) greater risk of hemorrhagic stroke. The ROS was 0.016, indicating a two-star rating. We did not find significant evidence of publication bias, and visual inspection of the funnel plot provides no evidence of substantial publication bias (Fig. 3c).

### Vegetable consumption and esophageal cancer

We identified six prospective cohort studies^35,36,55,56,59,62^ to evaluate the relationship between vegetable consumption and esophageal cancer, with a total sample size of 818,059 individuals. The follow-up duration ranged from 5 years to 16.3 years, with a median duration of 8.9 years. Three of the cohorts were from Asia^55,59,62^; two cohorts were from Europe^36,56^; and one cohort was from the United States^35^. Four of the cohorts used administrative medical records to assess the outcome^35,36,56,59^, whereas the other two cohorts used physician diagnosis^62^ or self-report^55^. Four of the cohorts used HRs^35,36,55,59^ as the effect size measure, and two of the cohorts used RRs^56,66^. All but one study adjusted the RR for smoking^35,36,55,56,59^.

**Fig. 4:**
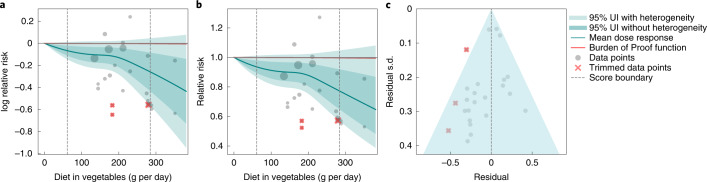
Vegetable consumption and esophageal cancer. **a**, log RR function. **b**, RR function. **c**, A modified funnel plot showing the residuals (relative to zero) on the *x* axis and the estimated s.d. that includes reported s.d. and between-study heterogeneity on the *y* axis.

We observed a statistically significant, non-linear monotonic decline in esophageal cancer risk with increasing vegetable consumption (Fig. 4). Uniquely among the outcomes included in this study, we observed modest risk reductions at lower consumption levels, with the steepest decline in risk occurring at high consumption levels. The mean risk of esophageal cancer at 100 g per day was 8.7% (95% UI including between-study heterogeneity = −0.005 to 16.4) lower than at 0 g per day, whereas it was just 3.7% (−0.002 to 7.2) lower at 200 g per day compared to 100 g per day; 14.3% (−0.01 to 26.2) lower at 300 g per day compared to 200 g per day; and 11.8% (−0.01 to 21.9) lower at 400 g per day compared to 300 g per day. Given the wide uncertainty around the estimated RR curve, especially at higher consumption levels, the nuances of the shape of the RR curve should be interpreted cautiously.

Compared to the TMREL, consuming no vegetables was associated with a 28.5% (−0.02 to 51.4) greater risk of esophageal cancer. The ROS was 0.001, corresponding to a two-star rating. We observed asymmetry in the funnel plot (Fig. 4c), and the result of the Egger’s regression suggested statistically significant evidence of small-study bias (Egger’s regression *P* = 0.031).

### Vegetable consumption and type 2 diabetes

We identified 15 prospective cohort studies^30,31,33,38,41,43–45,47,48,51,52,58,64,65^ and one case–cohort study^63^ evaluating the relationship between vegetable consumption and type 2 diabetes among 1,025,899 participants. Six cohorts^30,44,47,48,63,64^ were from Europe; five cohorts were from North America^31,43–45,51^; four cohorts were from Asia^33,41,58,65^; and two cohorts were from Australia^38,52^. Ten studies used self-report^38,41,43–45,51,52,58,64,65^ to assess outcomes; three studies used administrative medical records^47,48,63^; two studies used physician diagnosis^31,33^; and one study used biomarkers to assess the outcome of interest^30^. Follow-up time ranged from 4 years to 23 years (median: 10.6 years). In all studies, the effect size measure was adjusted for BMI, 12 studies adjusted for education^30,33,38,44,45,48,51,52,58,63–65^, and six studies adjusted for hypertension^30,33,41,43,58,65^. Eight studies reported effect sizes with HRs^30,31,33,48,51,58,63,65^; four studies reported ORs^38,41,44,64^; and the rest reported RR.

**Fig. 5:**
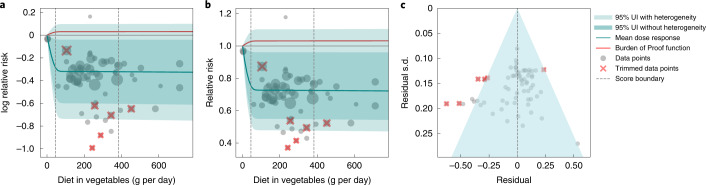
Vegetable consumption and type 2 diabetes. **a**, log RR function. **b**, RR function. **c**, A modified funnel plot showing the residuals (relative to zero) on the *x* axis and the estimated s.d. that includes reported s.d. and between-study heterogeneity on the *y* axis.

We observed a non-linear, monotonic decline in type 2 diabetes risk with increasing vegetable consumption that did not achieve statistical significance when accounting for between-study heterogeneity (Fig. 5). Compared to the TMREL, consuming no vegetables was associated with a 26.1% (−3.6 to 48.3) greater mean risk of type 2 diabetes. The exposure-averaged BPRF was opposite null from the mean risk curve and corresponded to an ROS of −0.031. The negative ROS equates to a one-star rating, indicating an association that does not achieve statistical significance after accounting for between-study heterogeneity. We did not find significant evidence of publication bias, and visual inspection of the funnel plot provides no evidence of substantial publication bias (Fig. 5c).

## Discussion

We applied our methodological framework^15^ both to quantify the association between vegetable consumption and five health outcomes and to assess the strength of the evidence for those associations. We found that increased vegetable consumption was associated with statistically significant reductions in the risk of ischemic stroke risk, and that, although evidence is suggestive of protective effects with type 2 diabetes, the evidence did not achieve statistical significance when between-study heterogeneity was accounted for. For all outcomes except esophageal cancer, we estimated that mean risk decreased non-linearly with increased consumption such that the most substantial declines occurred with increases from low to moderate consumption, and smaller marginal risk reduction occurred with increases from moderate to high consumption.

We found ROSs of 0.15 and 0.13 for ischemic stroke and IHD, respectively, corresponding to a three-star rating and a two-star rating for two outcomes (on a five-star scale), respectively. These ratings reflect the reasonably good quality and number of studies, large sample sizes and little between-study heterogeneity on the one hand and modest magnitude of the effect sizes on the other: all else being equal, risk–outcome pairs with weaker associations will have lower ROSs to reflect the greater potential for weak associations to result entirely from residual confounding or bias. For context, of the 180 risk–outcome pairs assessed with this method to date, 46 (25.6%) received a three-star rating; 112 (62.2%) received a rating of one or two stars; and only 22 (12.2%) received a rating of four or five stars^15^ (https://vizhub.healthdata.org/burden-of-proof). These findings suggest that we have stronger evidence to support the associations between vegetable consumption and both IHD and ischemic stroke than we do for most risk–outcome pairs in GBD but that the evidence is not overwhelming. Although the BPRF star and GRADE ratings are not directly comparable, it is notable that both approaches suggest that evidence with regard to IHD is moderately good but not of the highest quality^3^. Conversely, the two-star rating for IHD suggests weaker evidence than does the World Health Organization (WHO)/Food and Agriculture Organization (FAO) assessment of ‘convincing’ evidence for decreased risk of cardiovascular disease associated with fruits and vegetables, which corresponds to ‘evidence based on epidemiological studies showing consistent associations’^70^. This difference highlights the importance of considering the strength of the association and using a quantitative approach to assessing consistency (that is, between-study heterogeneity) when evaluating evidence.

Evidence for associations between vegetable consumption and hemorrhagic stroke and esophageal cancer is weaker, with each having a two-star rating. We found statistically significant evidence both with and without between-study heterogeneity for each of these two outcomes, but the strength of that evidence is weakened by the modest effect sizes, relatively limited number of studies and large between-study heterogeneity. The two-star rating for esophageal cancer is in line with, although perhaps somewhat stronger than, the assessment of the evidence as ‘limited, suggestive decreases risk’ by the World Cancer Research Fund and the American Institute for Cancer Research^71^. Evidence for an association between vegetable consumption and type 2 diabetes is still weaker, with a one-star rating. Although we found evidence trending toward a protective effect of some vegetable intake compared to none, the association did not achieve statistical significance using an approach to capturing uncertainty that accounts for between-study heterogeneity. For type 2 diabetes, we found statistically significant associations with vegetable consumption when not accounting for between-study heterogeneity, indicating that this lack of consistent findings across studies is a major factor underlying the weak ROS. The WHO and FAO did not include an assessment of the evidence for an association between vegetables in totality and type 2 diabetes; they assessed a protective effect of fruits and vegetables against type 2 diabetes as ‘probable’ based on evidence for non-starch polysaccharides, for which fruits and vegetables are a major source. The lack of clarity around vegetables and type 2 diabetes risk suggests a need for more research.

Our analysis suggests slightly, but not significantly, stronger protective effects of vegetables against IHD than reported in previous meta-analyses. For example, comparing 400 g to no vegetable consumption, the RR estimates from Gan et al.^1^ and the present study are 0.82 (95% CI: 0.73–0.92) and 0.76 (0.68–0.86), respectively; and comparing 200 g to no vegetable consumption, the RR estimates from Aune et al.^2^ and the present study are 0.84 (95% CI: 0.79–0.90) and 0.80 (0.72–0.88), respectively. Similarly, we estimated a stronger effect size with type 2 diabetes than did previous meta-analyses. Whereas Schwingshackl et al.^5^ estimated an RR of 0.92 (95% CI: 0.89–0.95) comparing consumption of 240 g per day versus 0 g per day and Halvorsen et al.^8^ estimated an RR 0.97 (95% CI: 0.94–1.01) comparing 200 g per day and 0 g per day, we estimated an RR of 0.73 (0.52–1.04). Note that, although our estimate suggests a stronger mean effect size, we also estimate much greater uncertainty, and our UIs include both previously published estimates. Previous meta-analyses of vegetable consumption and esophageal cancer have been mixed, and most estimated RRs by esophageal cancer type (that is, squamous cell carcinoma and adenocarcinoma), preventing direct comparison. Vingeliene et al.^9^ looked at vegetables and all esophageal cancer and estimated an RR of 0.98 (95% CI: 0.90–1.06) comparing 100 g to 0 g of vegetables daily, whereas we estimated a corresponding RR of 0.91 (0.84–1.00). As with the aforementioned outcomes, our estimates suggest a slightly, but not significantly, stronger effect size.

Although the methodological framework used in our work addresses many of the limitations of existing approaches, our study nonetheless has several important limitations. We identified no randomized trials of the effect of increased vegetable consumption on any of the five health outcomes. Because vegetable consumption is strongly associated with other aspects of diet and other demographic, social and behavioral risk factors (for example, age, income, education, physical activity and smoking)^72^, we expect strong confounding with any naturally observed association between vegetables and health in the absence of thoughtful and well-executed statistical controls. Broadly, we expect vegetable consumption to be positively associated with more healthful behaviors (for example, less smoking) and with demographic and social factors associated with improved health (for example, higher education levels). This suggests that confounding would distort the strength of the association upward (that is, higher observed RR). Although all of the studies in this analysis adjusted for some potential confounders, it is likely that some residual confounding remains due to imperfect statistical adjustment (for example, where continuous confounders are treated as categorical variables, some uncontrolled confounding will remain within each category, or where only a linear term is included in the model for confounders that have a non-linear association with disease risk); imperfect confounder measurement; and the existence of confounders that were unknown, unmeasured or otherwise unadjusted for. This is one reason that the BPRF methodology rates evidence of similar quality more strongly for risk–outcome pairs for which we observed a stronger association: strong associations are less likely to result entirely from residual confounding (and other sources of bias) than are weak associations^16^. Although we included bias covariates for imprecise exposure definitions, there are some potential sources of exposure misclassification for which we were unable to completely account: errors in measuring dietary intake are well-established due to self-reporting errors, random daily variation in diet and differences in perceptions of portion sizes^69^. Many studies are careful to define a serving size when collecting and reporting data; where studies did not define a serving size, however, we applied a consistent definition that could differ from perceptions or definitions of a serving size within the study context. For example, a dietary assessment calibration study in ten European countries found that participant definitions of a serving of vegetables ranged from 43.8 g in Sweden to 89.6 g in Greece^73^. Although random errors in exposure measurement will bias RR estimates toward the null, the effect of systematic errors are harder to predict. Finally, this study is subject to the limitations of the methodological framework^15^.

Based on our analysis, the evidence justifies more robust efforts and policies to promote increased vegetable consumption to reduce chronic disease risk, especially with regard to IHD, stroke and esophageal cancer. At the population level, increased vegetable consumption is also one component of a diet that is favorable for sustained planetary health. Given the large carbon footprint associated with the production of animal-based foods, *The Lancet* EAT Commission recommends that people consume a primarily plant-based diet, including 300 g of vegetables per day to promote both individual human health and environmental health^74^. Although our analyses did not evaluate the potential environmental benefits of increased vegetable consumption, our results do support the recommendation for increased vegetable consumption as it relates to individual-level health benefits, and our TMREL estimates of 306–372 g per day are in line with the Commission’s recommendation. Several other dietary guidelines express recommendations for fruits and vegetables combined: both the WHO and the World Cancer Research Fund recommend consuming at least 400 g of fruits and vegetables daily^70,75^, and the National Health Service of England’s Eat Well guide recommends at least 385 g of combined fruits and vegetables^76^. The United States Department of Agriculture’s *Dietary Guidelines for Americans* recommends 2.5 cups of vegetables daily, but both the volume-based measure and the inclusion of beans, lentils and starchy vegetables makes direct comparison to our estimates difficult^77^.

We found evidence that increasing vegetable consumption is associated with a reduction in IHD, ischemic stroke, hemorrhagic stroke and esophageal cancer risk, and we found a TMREL that is in line with existing recommendations for vegetable consumption. The ROSs for ischemic stroke and IHD, in particular, are among the highest for the dietary risk factors that have been evaluated to date, and the associated two-star and three-star ratings indicate that, even under a conservative interpretation of the evidence, vegetable consumption has a significant protective effect against ischemic stroke, IHD, hemorrhagic stroke and esophageal cancer risk. Although randomized controlled trials (RCTs) would help to definitively clarify the extent to which residual confounding and bias may distort estimates from observational studies, current evidence justifies more robust efforts and policies to promote increased vegetable consumption.

## Methods

### Overview

Using tools first published in Zheng et al.^16^, we applied the meta-analytic approach detailed in Zheng et al.^15^ to evaluate the magnitude and strength of evidence for the association between vegetable intake and five potential health outcomes. This approach can be summarized in six steps: (1) search for and extract data from published studies; (2) estimate the shape of the exposure-relative risk relationship; (3) test and adjust for biases across study designs and characteristics; (4) quantify unexplained between-study heterogeneity; (5) evaluate potential small-study effects and publication or reporting bias; and (6) estimate the BPRF—a quantification of the smallest harmful or protective effect (closest to null) across a range of exposures that is supported by the evidence—and use this estimate to compute the ROS and star rating on a scale of one to five. We describe these steps below, and further details are available in Zheng et al.^15^.

The estimates for our primary indicators from this work—RRs across a range of exposures, BPRFs, ROSs and star ratings for each risk–outcome pair—are not specific to or disaggregated by specific populations (that is, we did not estimate by location, sex or age group). The reports we referenced included information about the self-reported sex of the participants but did not all include sex-specific RR estimates. These factors precluded us from performing any sex-based or gender-based analyses.

We followed PRISMA guidelines^18^ (Supplementary Tables 1 and 2 and Extended Data Figs. 1–4) and Guidelines on Accurate and Transparent Health Estimate Reporting (GATHER) recommendations^78^ (Supplementary Table 4) for reporting our methods and analyses. This study was approved by the University of Washington institutional review board committee (study no. 9060) as a component of the GBD study. It was not registered as a systematic review.

### Conducting systematic reviews

We conducted systematic reviews to identify studies presenting relative measures of association (for example, RRs, ORs and HRs) between vegetable consumption and each of the five included health outcomes. We define vegetable consumption as the average daily consumption (in grams per day) of vegetables, including fresh, frozen, cooked, canned or dried vegetables, and excluding legumes and salted or pickled vegetables, juices, nuts and seeds and starchy vegetables, such as potatoes or corn. We defined our outcome as either incidence of, or mortality from, the health outcome, excluding studies that included other or non-specific outcome definitions (for example, unspecified cardiovascular disease).

We first searched the most recent PRISMA-compliant meta-analyses that examined associations between vegetable consumption and IHD^79^, stroke subtypes^79^ and type 2 diabetes^8^. Search strings were developed to identify the meta-analyses for each outcome. We searched the citations of each identified meta-analysis. A separate search string was developed to identify sources published after the period covered in the identified most recent PRISMA-compliant meta-analysis for each outcome of interest. We searched PubMed, EMBASE and Web of Science from the last date of the identified meta-analysis through 31 May 2022. We also searched the Global Health Data Exchange (GHDx) databases. For vegetable consumption and esophageal cancer, we conducted a full systematic review with no date constraint because we did not find a meta-analysis that fulfilled our selection criteria (PRISMA-complaint and matching our definition of exposure and outcome). A detailed description of the search strings and searching strategy is presented in the Supplementary Appendix. After removing duplicate search results, reviewers manually screened each study for eligibility on the basis of title and abstract. As a sensitivity check, 20% of title and abstract exclusions by each reviewer were verified by a second reviewer. The full text of the remaining studies was retrieved and examined for inclusion in this analysis. Finally, each full-text exclusion was verified by both reviewers. Only one discrepancy was found and was settled by moving to full-text review for additional evaluation.

Studies were eligible for inclusion if they used suitable exposure and outcome definitions, provided some measure of uncertainty (for example, sample size, standard error or CIs) and reported an RR (or related measure) for which the exposed and unexposed groups were defined. Studies that estimated associations with a select subset of vegetables (for example, green/yellow vegetables, cruciferous vegetables and alliums) were excluded. Where multiple studies provided RR estimates from the same cohort, we included only the study that captured the largest sample or follow-up time so as not to include duplicate data. For each study, two reviewers manually extracted data on study name, location, design, population (age, sex, race and sample size), duration of follow-up, exposure definition, exposure assessment method, exposure categories, outcome definition, outcome ascertainment method and covariates included in the statistical analysis of the study. All included studies published the required data, and no unpublished data were obtained for this analysis. For each exposure category, we also collected data on the range of exposure, number of participants, person-years, number of events and risk estimate and its corresponding uncertainty. See Supplementary Table 5 for a full list of extracted variables. We standardized the exposure unit to grams (g) of consumption per day. For studies reporting the consumption in servings of vegetables with no other corresponding information about the serving size, we assumed a serving size of 77 g per day^79,80^. For studies that reported mean consumption rather than ranges of vegetable consumption, we used the midpoint between means as the cutoff for intake intervals. For undefined lower bounds, we assumed a consumption level of 0 g per day. For undefined upper bounds when the mean and standard deviation values were not available, we applied the range from the cohort’s most adjacent quartile or tertile to estimate the upper bound of consumption, specific to each study cohort. All studies included in the analysis for each risk–outcome pair and study characteristics are given in Supplementary Table 3.

### Estimating the shape of the exposure-relative risk relationship

We modeled a mean RR (a measure of effect size) curve across the range of exposure for each risk–outcome pair using MR-BRT, a Bayesian meta-regression tool detailed previously^16^. MR-BRT has capabilities that are useful for estimating risk curves and assessing the quality of evidence for the modeled association, including (1) the ability to model non-linear associations with splines with automatic knot selection and shape constraints; (2) the ability to use study-level covariates to account for elements of study design that could introduce bias; (3) the ability to estimate uncertainty with and without consideration of between-study heterogeneity; (4) the ability to adjust the between-study heterogeneity parameter to penalize data sparsity through a Fisher scoring correction; and (5) the ability to assess publication bias^15^.

We followed a consistent approach to choosing model specifications for all dietary risks. We first modeled the association with no constraints to assess the nature of the association and classified risk curves as protective (risk declining with increasing exposure), harmful (risk increasing with increasing exposure) or J-shaped (risk increasing with exposure levels above or below a global minimum). We then applied the same number of spline degrees and knots and same priors and constraints to all risk–outcome pairs with a given shape. In the case of vegetable consumption and each of the five outcomes presented here, our unconstrained models suggested that disease risk declined with increasing vegetable consumption, corresponding to a protective risk. As a protective risk, we ran the final models using quadratic splines with two internal knots, a monotonic decreasing prior and a linearity prior on the right tail. Major components of the meta-regression model were validated before their use in this study, including simulating scenarios for (1) many studies with many data points per study; (2) many studies with few data points per study; and (3) few studies with few data points per study. For each simulation, results from the approach used here were compared with results from three existing approaches: (1) a log-linear meta-analysis implemented in the meta-for package^81^; (2) a one-stage approach for dose–response meta-analysis^82^, implemented in the dosremeta package^83^; and (3) a two-stage approach for dose–response meta-analysis^84^, implemented in the dosremeta package^83^. The approach used here performed as well as or better than existing approaches across all three scenarios, particularly for non-linear relationships^15^.

### Testing and adjusting for biases across study designs and characteristics

For each study reporting an effect size for the association between vegetable consumption and the included outcomes, we extracted information about aspects of study design that could potentially bias the reported effect size and coded this information into study-level covariates. These study-level covariates included follow-up period (≤10 months and >10 months), precision of the exposure and outcome definitions, study design (that is, RCT or cohort study), reported measure of association (RRs or ORs), included outcome events (incidence or mortality), number of exposure measurements (single or repeat), method by which outcomes were ascertained (administrative records or self-reports) and the level of adjustment for relevant confounders, such as age, sex, smoking, education, calorie intake and income. We adjusted for these covariates in our meta-regression if they significantly biased our estimated RR function^15^. See Supplementary Table 6 for results from our assessment of study quality for all included studies.

### Evaluating between-study heterogeneity, uncertainty and small numbers of studies

Heterogeneity in RR estimates among different studies may reflect methodological differences, random variation or true inconsistencies in effect sizes in different study populations. We used the aforementioned study-level covariates to account for between-study heterogeneity that results from differences in study design and used a linear mixed-effects model to capture the remaining unexplained between-study heterogeneity^15^. As described in Zheng et al.^15^, we implemented the Fisher information matrix to the heterogeneity parameter, which corrects potential underestimation of between-study heterogeneity for data-sparse situations. In such cases, the between-study heterogeneity parameter estimate may be zero, simply from lack of data. The Fisher information matrix uses a quantile of gamma, which is sensitive to the number of studies, study design and reported uncertainty. We calculated 95% UIs for each mean risk curve both with between-study heterogeneity incorporated (a ‘conservative’ UI) and without it incorporated (a ‘conventional’ UI), but we present only the UIs that include between-study heterogeneity.

### Evaluating potential for publication or reporting bias

Egger’s regression assesses small-study bias by estimating the association between model residuals and the residual standard deviation of the corresponding data points, and the *P v*alue to assess the statistical significance of that association, where the residual standard deviation incorporates both the standard error of the data point and between-study heterogeneity^85^. Small-study biases may arise from random change (that is, more heterogeneity due to random chance with small samples), differences in study quality or analysis or publication and reporting biases. The formal statistical test used here decreases the chance of flagging apparent small-study biases that arise only from random chance; and our application of bias covariates in the RR model decreases the change of systematic differences in model residuals arising from differences in study quality or analysis. In this context, Egger’s regression becomes a tool primarily to test for empirical evidence of potential publication or reporting biases^85^ that identifies significant evidence of publication bias as a statistically significant association between model residuals and the residual standard deviations of the corresponding data points. Significant results for Egger’s regression are flagged as indicating a potential risk for publication and/or reporting bias^15^.

### Estimating the TMREL/minimum risk exposure level

The TMREL is the level of exposure that, within the range of values that are theoretically possible at the population level, will minimize the risk of all outcomes associated with that risk combined. For harmful exposures that may be theoretically eliminated, the TMREL is typically zero (for example, smoking); and for exposures with U-shaped risk curves, the TMREL is typically set to the exposure level that corresponds to the minimum of the risk curve (for example, alcohol). Defining the TMREL for protective exposures, however, is more conceptually difficult and requires us to consider three factors: 1) we cannot assume that marginal protective effects persist with increases in exposure beyond the exposure levels that we observe in the source data (that is, there may plausibly be an exposure level beyond which benefits reverse, with a true minimum risk level occurring outside the range of observed data); 2) for most protective exposures, the risk curve will be steepest at lower levels of exposure and flatten at higher exposure levels, leading to risk reductions at higher levels that are neither statistically nor clinically significant; and 3) as we move toward very high exposure levels, the requirement for the TMREL to be theoretically possible becomes relevant and supports basing the TMREL on plausibility. We, therefore, selected the TMREL based on exposure levels reported in the included RR studies. For each study, we took the range of vegetable consumption for the highest consumption group. We defined the lower bound of the TMREL as the 85th percentile of the lower limit of that range across all studies, and we defined the upper bound of the TMREL as the 85th percentile of the midpoint of the range across all studies. This approach yields a TMREL that corresponds to high real-world consumption levels for which we have adequate data to draw robust conclusions about risk.

### Estimating the BPRF

Using the risk curve modeled with the MR-BRT tool and incorporating between-study heterogeneity into our estimate of uncertainty, we estimated the BPRF as the function that corresponds to the 5th (for harmful risks) or 95th (for protective risks) quantile of the log RR curve that is closest to the null. The BPRF represents the weakest association that is consistent with the available data after accounting for between-study heterogeneity, and the further the BPRF falls from the null, the stronger the evidence is for that association^15,16^. We then estimated the ROS as the mean value of the log-BPRF averaged over the 15th and 85th percentiles of the distribution of vegetable consumption observed in the studies used to estimate the association between vegetable consumption and the outcome of interest. Because log RRs are symmetric about zero, with harmful risks having positive values for the log RR and protective effects having negative values, we multiplied the ROS by –1 for each risk–outcome pair in this analysis, because vegetable intake is a protective risk. The ROS, therefore, provides a single summary of the log RR that is comparable across both protective and harmful effects. A higher positive ROS corresponds to a stronger association, whereas a negative ROS indicates that the available evidence did not reject the null hypothesis. Finally, we converted the ROS for each risk–outcome pair into a star rating on a scale of one to five, with a one-star rating indicating a non-significant relationship based on the conservative interpretation and two-star through five-star ratings representing an increase in risk with average exposure (compared to no exposure) in the ranges of 0–15% for two-star pairs, >15–50% for three-star pairs, >50–85% for four-star pairs and greater than 85% for five-star pairs for harmful risks and a decrease in risk with average exposure (compared to no exposure) in the ranges of 0–13% for two-star pairs, >13–34% for three-star pairs, >34–46% for four-star pairs and greater than 46% for five-star pairs for protective risks^15^.

### Sensitivity analyses

For each risk–outcome pair, we conducted sensitivity analyses that compared the dose–response curves generated with and without trimming the 10% least coherent data points (online methods section 8 in Zheng et al.^15^). We also conducted additional sensitivity analysis by including calorie intake adjustment as a pre-selected bias covariate. The dose–response curves of the sensitivity analyses are shown in Supplementary Figs. 1–6.

### Statistical analysis

Analyses were performed using R version 3.6.1, Python version 3.8 and Stata version 17.

### Statistics and reproducibility

This study was a secondary analysis of existing data involving systematic reviews and meta-analyses. No statistical method was used to pre-determine sample size. As the study did not involve primary data collection, randomization, blinding and data exclusions are not relevant to this study and, as such, no data were excluded, and we performed no randomization or blinding. We have made our data and code available to foster reproducibility.

### Reporting summary

Further information on research design is available in the Nature Research Reporting Summary linked to this article.

## Online content

Any methods, additional references, Nature Research reporting summaries, source data, extended data, supplementary information, acknowledgements, peer review information; details of author contributions and competing interests; and statements of data and code availability are available at 10.1038/s41591-022-01970-5.

## Supplementary information

Supplementary Information

Reporting Summary

## Data Availability

The findings from this study were produced using data available in public online repositories and in the published literature. Data sources and citations for each risk–outcome pair can be downloaded using the ‘download’ button on each risk curve page available at https://vizhub.healthdata.org/burden-of-proof/. Citations for all input studies are listed in the main text reference list as refs. ^17–66^. Study characteristics for all input data used in the analyses are also provided in Supplementary Table 3. See Supplementary Table 5 for a template of the data collection form.

## Code availability

All code used for these analyses is publicly available online (https://github.com/ihmeuw-msca/burden-of-proof). This includes code for the meta-regression engine, the model specification interface and both parts of the data processing.
