# Supplementary material for: Health effects associated with vegetable consumption: a Burden of Proof study
Source: Nat Med. 2022 Oct 10;28(10):2066–74. doi: 10.1038/s41591-022-01970-5 (PMC9556321; doi:10.1038/s41591-022-01970-5)
Supplement: Supplementary file 1 — Supplementary Information [file 41591_2022_1970_MOESM1_ESM.pdf]

nature portfolio

https://doi.org/10.1038/s41591-022-01970-5

Supplementary information

Health effects associated with vegetable consumption: a Burden of Proof study

In the format provided by the authors and unedited

Supplementary Information: supplementary methods, data sources, and results for “Health effects associated with vegetable consumption: a Burden of Proof study"

This appendix provides detailed information on input data sources, and presents supplementary results—particularly sensitivity analysis results—for “Health effects associated with vegetable consumption: a Burden of Proof study."

Contents

Section 1: Data source identification and assessment 3
Section 1.1: Literature identification 3
Section 1.2: Assessing data source eligibility 6
Section 2. PRISMA checklists 7
Supplementary Table 1. PRISMA 2020 checklist 7
Supplementary Table 2. PRISMA 2020 abstract checklist 9
Section 3: Study characteristics 10
Supplementary Table 3. Studies characteristics 10
Section 4. GATHER checklists 22
Supplementary Table 4. GATHER checklist 22
Section 5: Causal criteria 22
Supplementary Table 5. Causal criteria extraction template 23
Section 6: Study quality and risk of bias assessment 28
Supplementary Table 6. Study quality for every study used in the models 28
Section 7: Results from individual studies 30
Supplementary Table 7. Summary results from input studies 30
Section 8: Sensitivity results 37
Supplementary Figure 1: Vegetable consumption and ischemic stroke: Non-trimmed data. 37
Supplementary Figure 2: Vegetable consumption and ischemic heart disease: Non-trimmed data. 37
Supplementary Figure 3: Vegetable consumption and hemorrhagic stroke: Non-trimmed data. 38
Supplementary Figure 4: Vegetable consumption and esophageal cancer: Non-trimmed data. 38
Supplementary Figure 5: Vegetable consumption and type 2 diabetes: Non-trimmed data. 39
Supplementary Figure 6: caloric adjusted vs unadjusted 40

Section 1: Data source identification and assessment

The data used for this study can be categorized into the following types: prospective cohort and case-cohort. Underlying data and citations are available for download using the “download” button on each risk curve page at https://vizhub.healthdata.org/burden-of-proof/.

Section 1.1: Literature identification

We conducted literature searches to obtain input data from prospective studies evaluating the relationship between vegetable consumption and ischemic heart disease (IHD), ischemic stroke, hemorrhagic stroke, type 2 diabetes, and esophageal cancer in our analysis.

A search string was developed to identify the most recent PRISMA-compliant meta-analyses which examined the effect of vegetable consumption and IHD, ischemic stroke, hemorrhagic stroke, and type 2 diabetes. Then the citation of the selected meta-analysis was searched. After selecting a meta-analysis for each outcome, another search string was developed to identify sources published after the period covered in the identified most recent PRISMA-compliant meta-analysis for each outcome of interest. For each of the outcome of interests, we searched PubMed, EMBASE, and Web of Science from the last date of the identified meta-analysis through May 31, 2022. We also searched the global health data exchange (GHDx) databases. For vegetable consumption and esophageal cancer, we conducted a full systematic review with no date constraint because we did not find a meta-analysis that meets our selection criteria. A full description of the search strings and searching strategy is presented below.

Ischemic Heart diseases: We developed PubMed strings to identify the most recent PRISMA complaint meta-analysis on vegetable consumption and IHD. The most recent PRISMA complaint meta-analysis that met our definition of exposure (i.e., vegetable consumption) and outcome (i.e., Ischemic heart diseases) was a study published by Bechtold et al in 2019. From this study meta-analysis, we identified 32 citations, with 11 studies meeting the inclusion criteria to be included in the final analysis. We found three additional studies from the global health data exchange (GHDX) database but only two studies met our inclusion criteria.

Search strings for ischemic heart diseases	***Identified meta-analysis***
("Vegetables"[Mesh] OR Vegetables [Title/Abstract] OR "green leafy vegetables "OR" Cruciferous vegetables "OR" Fruits and Vegetables" [Title/Abstract]) AND ("Systematic Review" [Publication Type] OR "systematic review" [Title/Abstract] OR "Meta-Analysis" [Publication Type] OR "meta-analysis" OR "Dose-Response"[Title/Abstract]) AND ("Myocardial Ischemia" [Mesh] OR "Coronary Artery Disease" [Mesh] OR "Angina, Stable" [Mesh] OR "Acute Coronary Syndrome" [Mesh] OR "ischemic heart disease" [tiab] OR "ischaemic heart disease" [tiab] OR "coronary artery disease" [tiab] OR "coronary heart disease" [tiab] OR "myocardial ischemia" [tiab] OR "myocardial ischaemia" [tiab] OR "myocardial infarction" [tiab] OR "angina" [tiab] OR "acute coronary syndrome")	Angela Bechthold, Heiner Boeing, Carolina Schwedhelm, Georg Hoffmann,Sven Knüppel, Khalid Iqbal, Stefaan De Henauw, Nathalie Michels, Brecht Devleesschauwer,Sabrina Schlesinger & Lukas Schwingshackl (2019) Food groups and risk of coronary heart disease, stroke and heart failure: A systematic review and dose-response meta-analysis of prospective studies, Critical Reviews in Food Science and Nutrition, 59:7, 1071-1090.

A search string was developed to identify sources published after the period covered in Bechtold (March 1, 2017 to May 31, 2022). PubMed, EMBASE, and Web of Science were searched on May 31, 2022. The searches returned 473 total citations. Duplicates between the search results were identified by DOI and PubMed, then the remaining records were screened by title and abstract for possible retrieval. As a sensitivity test, 20% of each reviewer’s exclusions were independently verified by the other reviewer. The full text of included studies was retrieved and evaluated for extraction. Each full-text exclusion was verified by both reviewers. No discrepancies occurred

between the 2 reviewers. Each exclusion reason source count is depicted in the Vegetable-IHD PRISMA diagram (Extended Data Figure 2).

Search Strings:

("Vegetables"[Mesh] OR "Vegetables" [Title/Abstract] OR "green leafy vegetables" OR "leafy vegetables" OR "Cruciferous vegetables" OR "Fruits and Vegetables" [Title/Abstract])
AND ("Cohort" [Publication type] OR "Prospective cohort" [Title/Abstract] OR "case-cohort" OR "Follow-up" OR "Longitudinal" [Title/Abstract])
AND ("Myocardial Ischemia" [Mesh] OR "Coronary Artery Disease" [Mesh] OR "Angina, Stable" [Mesh] OR "Acute Coronary Syndrome" [Mesh] OR "ischemic heart disease" [tiab] OR "ischaemic heart disease" [tiab] OR "coronary artery disease" [tiab] OR "coronary heart disease" [tiab] OR "myocardial ischemia" [tiab] OR "myocardial ischaemia" [tiab] OR "myocardial infarction" [tiab] OR "angina" [tiab] OR "acute coronary syndrome")

Stroke subtypes (Ischemic and hemorrhagic stroke). We developed search strings for both stroke subtypes (i.e. hemorrhagic stroke and ischemic strokes) to identify the most recent PRISMA complaint meta-analyses that fulfilled our definition of exposure (total vegetable consumption) and outcomes (stroke subtypes: Ischemic and hemorrhagic stroke). The meta-analysis used for IHD (Bechtold et al 2019) was determined to be the best source available for stroke citations as well. Due to the substantial overlap among studies on ischemic and hemorrhagic stroke, sources were screened simultaneously for data on either outcome. A total of 38 citations were identified in the citations of this meta-analysis and screened. From a total of 38 citations, only eight studies (all eight studies included ischemic stroke while 5 of them included hemorrhagic stroke only) were eligible to be included in the final analysis. From the GHDX database, we identified three studies and only two of these studies met our inclusion criteria (reported both Ischemic and hemorrhagic stroke).

**Search strings for hemorrhagic stroke**	**Identified meta-analysis**
("Vegetables"[Mesh] OR Vegetables [Title/Abstract] OR "green leafy vegetables "OR" Cruciferous vegetables "OR" Fruits and Vegetables" [Title/Abstract]) AND ("Systematic Review" [Publication Type] OR "systematic review" [Title/Abstract] OR "Meta-Analysis" [Publication Type] OR "meta-analysis" OR "Dose-Response"[Title/Abstract]) AND ("Hemorrhagic Stroke" [Mesh] OR ("subarachnoid" [Title/Abstract] AND ("hemorrhage" [Title/Abstract] OR "haemorrhage" [Title/Abstract])) OR "stroke" [Title/Abstract])	Angela Bechthold, Heiner Boeing, Carolina Schwedhelm, Georg Hoffmann,Sven Knüppel, Khalid Iqbal, Stefaan De Henauw, Nathalie Michels, Brecht Devleesschauwer,Sabrina Schlesinger & Lukas Schwingshackl (2019) Food groups and risk of coronary heart disease, stroke and heart failure: A systematic review and dose-response meta-analysis of prospective studies, Critical Reviews in Food Science and Nutrition, 59:7, 1071-1090.

**Search strings for ischemic stroke**	**Identified meta-analysis**
("Vegetables"[Mesh] OR Vegetables [Title/Abstract] OR "green leafy vegetables "OR" Cruciferous vegetables "OR" Fruits and Vegetables" [Title/Abstract]) AND ("Systematic Review" [Publication Type] OR "systematic review" [Title/Abstract] OR "Meta-Analysis" [Publication Type] OR "meta-analysis" OR "Dose-Response"[Title/Abstract]) AND ("Ischemic Stroke" [Mesh] OR "ischemic stroke" [Title/Abstract] OR "ischaemic stroke" [Title/Abstract] OR "cerebral infarction" [Title/Abstract] OR "unspecified stroke" [Title/Abstract] OR "stroke" [Title/Abstract])	Angela Bechthold, Heiner Boeing, Carolina Schwedhelm, Georg Hoffmann,Sven Knüppel, Khalid Iqbal, Stefaan De Henauw, Nathalie Michels, Brecht Devleesschauwer,Sabrina Schlesinger & Lukas Schwingshackl (2019) Food groups and risk of coronary heart disease, stroke and heart failure: A systematic review and dose-response meta-analysis of prospective studies, Critical Reviews in Food Science and Nutrition, 59:7, 1071-1090.

A search string was developed to identify sources published after the period covered in Bechtold et al 2019 study. PubMed, EMBASE, and Web of Science were searched on May 31, 2022. The search was filtered for publication dates beginning March 01, 2017, the cutoff date for the search in Bechtold et al 2019 study. The search string included terms for each stroke subtype, and sources were screened simultaneously for both stroke subtypes of interest. The searches returned 356 total citations, of which 2 records were ultimately extracted and included in the model. As described in the section on IHD, search results were deduplicated and screened by two reviewers, with each reviewer verifying the other's exclusions for both the title-abstract and full-text screening steps.

Search strings for stroke subtypes:

("Vegetables"[MeSH Terms] OR "Vegetable"[Title/Abstract] OR "green leafy vegetable*"[All Fields] OR "leafy vegetable*"[All Fields] OR "Cruciferous vegetables"[All Fields] OR "Fruits and Vegetables"[Title/Abstract] OR "Fruit and Vegetable"[Title/Abstract])*
AND ("Cohort studies" [Mesh] OR "Prospective cohort" [Title/Abstract] OR "case-cohort" OR "Follow-up" OR "Longitudinal" [Title/Abstract])
AND (("Ischemic Stroke" [Mesh] OR "ischemic stroke" [Title/Abstract] OR "ischaemic stroke" [Title/Abstract] OR "cerebral infarction" [Title/Abstract] OR "unspecified stroke" [Title/Abstract] OR "stroke" [Title/Abstract])
OR ("Hemorrhagic Stroke" [Mesh] OR "Intracranial Hemorrhages"[Mesh] OR "intracerebral hemorrhage"[Title/Abstract] OR "intracerebral haemorrhage"[Title/Abstract] OR "hemorrhagic stroke"[Title/Abstract] OR "haemorrhagic stroke"[Title/Abstract] OR ("subarachnoid" [Title/Abstract] AND ("hemorrhage" [Title/Abstract] OR "haemorrhage" [Title/Abstract]))))).

Diabetes: We developed a PubMed search string to identify the most recent PRISMA complaint meta-analysis on vegetable consumption and type 2 diabetes. The meta-analysis identified that fulfills our criteria of exposure definition (total vegetables consumption), outcome definition (Type 2 diabetes) and PRISMA complaint was *Halvorsen et al 2021*. A total of 51 citations of this meta-analysis were identified and screened. From this meta-analysis, we included a total of 13 studies in the final analysis. The last date this meta-analysis covered was October 20, 2020.

Search strings for diabetes	Identified meta-analysis
"Vegetables"[Mesh] OR Vegetables [Title/Abstract] OR "green leafy vegetables "OR" Cruciferous vegetables "OR" Fruits and Vegetables" [Title/Abstract]) AND ("Systematic Review" [Publication Type] OR "systematic review" [Title/Abstract] OR "Meta-Analysis" [Publication Type] OR "meta-analysis" OR "Dose-Response"[Title/Abstract]) AND ("Diabetes Mellitus, Type 2" [Mesh] OR "diabetes mellitus type 2" [Title/Abstract] OR "Gestational diabetes" OR "diabetes type 2" [Title/Abstract] OR "type 2 diabetes mellitus" [Title/Abstract] OR "type 2 diabetes" [Title/Abstract] OR "non-insulin dependent diabetes" [Title/Abstract] OR "adult-onset diabetes" [Title/Abstract])).	Rine Elise Halvorsen, Mathilde Elvestad, Marianne Molin, Dagfinn Aune. Fruit and vegetable consumption and the risk of type 2 diabetes: a systematic review and dose-response meta-analysis of prospective studies. BMJ Nutr Prev Health. 2021 Jul 2;4(2):519-531. doi: 10.1136/bmjnph-2020-000218.

Another search string was developed to identify sources published after the period covered in Halvorsen et al 2021. PubMed, EMBASE, and Web of Science and searched on May 31, 2022. The searches from the three databases returned 262 total hits, of which 3 studies were ultimately extracted and included in the model. As with the other risk-outcome reviews, after deduplication, two reviewers screened the results independently, with all full-text exclusions and 20% of title-abstract exclusions verified by both reviewers. The exclusion reasons by source count were reported in the PRISMA diagram (Extended Data Figure 4**).** We also searched the global health data exchange database to identify studies not caught from the searching of citation of the selected meta-analysis and the three databases.

Search strings:

("Vegetables"[Mesh] OR "Vegetables" [Title/Abstract] OR "green leafy vegetables" OR "leafy vegetables" OR "Cruciferous vegetables" OR "Fruits and Vegetables" [Title/Abstract])
AND ("Cohort" [Publication type] OR "Prospective cohort" [Title/Abstract] OR "case-cohort" OR "Follow-up" OR "Longitudinal" [Title/Abstract])
AND ("Diabetes Mellitus, Type 2" [Mesh] OR "diabetes mellitus type 2" [Title/Abstract] OR "diabetes type 2" [Title/Abstract] OR "type 2 diabetes mellitus" [Title/Abstract] OR "type 2 diabetes" [Title/Abstract] OR "non-insulin dependent diabetes" [Title/Abstract] OR "adult-onset diabetes" OR "Diabetes Mellitus" OR "T2D"[Title/Abstract])

Esophageal cancer: We conducted a full systematic review with no date constraint because we did not find a meta-analysis that fulfilled our selection criteria (I.e. PRISMA complaint and matching our definition of exposure and outcome). A search string was developed to identify all sources published with no date constraint. PubMed, EMBASE, and Web of Science were searched on June 16, 2022. The searches returned 214 citations, and after deduplication by DOI and PMID, there were 151 unique records. Each record was screened on title and abstract by one reviewer, finding 20 inclusions. As a sensitivity check, 20% of the 133 exclusions were validated by a second reviewer. 1 discrepancy was found and was settled by moving to full-text review for additional evaluation. During the full text review of the 20 sources, 6 met inclusion criteria and 14 were excluded. Each full-text exclusion was verified by both reviewers. Exclusion reasons by source count described in the PRISMA flow chart (Extended Data Figure 3).

Vegetable and esophageal cancer search strings:

("Vegetables"[Mesh] OR "Vegetables" [Title/Abstract] OR "green leafy vegetables" OR "leafy vegetables" OR "Cruciferous vegetables" OR "Fruits and Vegetables" [Title/Abstract])

AND ("Cohort" [Publication type] OR "Prospective cohort" [Title/Abstract] OR "case-cohort" OR "Follow-up" OR "Longitudinal" [Title/Abstract])

AND ("Esophageal cancer" [Mesh] OR "Esophageal cancer" [Title/Abstract] OR "Esophageal squamous cell carcinoma" [Title/Abstract] OR "esophageal adenocarcinoma" [Title/Abstract]

Section 1.2: Assessing data source eligibility

See Extended Data Figures 1-4 for details on identifying, screening, and assessing eligibility for records identified through our search.

Inclusion criteria

- Reported a relative risk of total vegetable consumption and at least one of the five outcomes
- Included a measure of uncertainty for the effect size measure
- Quantified the amount of vegetable consumption in the reference and alternate group
- Prospective cohort study or Nested case-control or case cohort study

Exclusion criteria

- Were an aggregate study: meta-analysis or pooled cohort
- Wrong study type: not a cohort study or case-cohort study
- Duplicate study: cohort reported in paper was also reported elsewhere
- Unmeasurable exposure: reported vegetable consumption without grams or servings equivalent, such as in aggregated "diet scores"
- No measure of interest: reported RR for change in vegetable consumption or does not report RR
- No exposure of interest: did not report any vegetable exposure or only reported a specific vegetable subtype
- No outcome of interest: reported on all-cause-mortality or an outcome outside of the five outcomes of interest studied in this paper. This includes outcomes lacking specificity such as total stroke or cardiovascular disease
- Not in English
- Non-general population: study population defined by comorbidity or other traits that could interact with exposure and affect outcome

For reports that met the inclusion criteria, data were extracted for the variables listed in Supplemental Table 4.

Section 2. PRISMA checklists

Supplementary Table 1. PRISMA 2020 checklist

Section and Topic	Item #	Checklist item	Location where item is reported
**TITLE**			
Title	1	Identify the report as a systematic review.	Per the journal's request, the title does not include "systematic review". It is, however, in the title of the methods section, "conducting systematic reviews"
**ABSTRACT**			
Abstract	2	See the PRISMA 2020 for Abstracts checklist.	See PRISMA 2020 for Abstracts Checklist below (Supplementary Table 2)
**INTRODUCTION**			
Rationale	3	Describe the rationale for the review in the context of existing knowledge.	"Main" (intro) paragraphs 1–3
Objectives	4	Provide an explicit statement of the objective(s) or question(s) the review addresses.	"Main" (intro) paragraph 4
**METHODS**			
Eligibility criteria	5	Specify the inclusion and exclusion criteria for the review and how studies were grouped for the syntheses.	Criteria summarized in Methods section "data"; full inclusion and exclusion criteria listed in SI section 1.2; reasons for exclusion and number of studies excluded also provided in PRISMA flow diagram for each pair (Extended Data Figures 1-4)
Information sources	6	Specify all databases, registers, websites, organisations, reference lists and other sources searched or consulted to identify studies. Specify the date when each source was last searched or consulted.	Methods section "conducting systematic reviews": paragraph 2; SI section 1.1
Search strategy	7	Present the full search strategies for all databases, registers and websites, including any filters and limits used.	Methods section "conducting systematic reviews": paragraph 2; SI section 1.1
Selection process	8	Specify the methods used to decide whether a study met the inclusion criteria of the review, including how many reviewers screened each record and each report retrieved, whether they worked independently, and if applicable, details of automation tools used in the process.	Methods section "conducting systematic reviews" paragraph 2-3
Data collection process	9	Specify the methods used to collect data from reports, including how many reviewers collected data from each report, whether they worked independently, any processes for obtaining or confirming data from study investigators, and if applicable, details of automation tools used in the process.	Methods section "conducting systematic reviews" paragraph 2-3
Data items	10a	List and define all outcomes for which data were sought. Specify whether all results that were compatible with each outcome domain in each study were sought (e.g. for all measures, time points, analyses), and if not, the methods used to decide which results to collect.	Methods sections "conducting systematic reviews"
	10b	List and define all other variables for which data were sought (e.g. participant and intervention characteristics, funding sources). Describe any assumptions made about any missing or unclear information.	Methods section "data" paragraph 2; full list and definitions of all variables are in Supplemental Table 5; study characteristics for each included study are also listed in Supplemental Table 3
Study risk of bias assessment	11	Specify the methods used to assess risk of bias in the included studies, including details of the tool(s) used, how many reviewers assessed each study and whether they worked independently, and if applicable, details of automation tools used in the process.	Methods section "testing and adjusting for biases across study designs and characteristics"
Effect measures	12	Specify for each outcome the effect measure(s) (e.g. risk ratio, mean difference) used in the synthesis or presentation of results.	Method sections "overview" paragraph 2, "estimating the burden of proof risk function"
Synthesis methods	13a	Describe the processes used to decide which studies were eligible for each synthesis (e.g. tabulating the study intervention characteristics and comparing against the planned groups for each synthesis (item #5)).	Methods section "data"

	13b	Describe any methods required to prepare the data for presentation or synthesis, such as handling of missing summary statistics, or data conversions.	Methods section “conducting systematic reviews”
	13c	Describe any methods used to tabulate or visually display results of individual studies and syntheses.	Methods sections “conducting systematic reviews,” “estimating the shape of the risk-outcome relationship”
	13d	Describe any methods used to synthesize results and provide a rationale for the choice(s). If meta-analysis was performed, describe the model(s), method(s) to identify the presence and extent of statistical heterogeneity, and software package(s) used.	Methods sections “Estimating the shape of the risk-outcome relationship,” “estimating the TMREL/minimum risk exposure level,” and “estimating the burden of proof risk function”. Software packages described in “code availability” section of the manuscript
	13e	Describe any methods used to explore possible causes of heterogeneity among study results (e.g. subgroup analysis, meta-regression).	Methods section “evaluating between-study heterogeneity, uncertainty, and small numbers of studies”
	13f	Describe any sensitivity analyses conducted to assess robustness of the synthesized results.	Methods section “sensitivity analyses”; SI section 8
Reporting bias assessment	14	Describe any methods used to assess risk of bias due to missing results in a synthesis (arising from reporting biases).	Methods for detecting publication or reporting bias found in methods section “evaluating potential for publication or reporting bias”
Certainty assessment	15	Describe any methods used to assess certainty (or confidence) in the body of evidence for an outcome.	Methods section “evaluating between-study heterogeneity, uncertainty, and small numbers of studies”
**RESULTS**			
Study selection	16a	Describe the results of the search and selection process, from the number of records identified in the search to the number of studies included in the review, ideally using a flow diagram.	PRISMA flow diagram for each risk-outcome pair (Extended Data Figures 1-4); first paragraph of each risk-outcome pair results section + the results “overview”
	16b	Cite studies that might appear to meet the inclusion criteria, but which were excluded, and explain why they were excluded.	N/A
Study characteristics	17	Cite each included study and present its characteristics.	SI section 3, Supplemental Table 3 (“study characteristics”); citations also available for download from the online viz tools: (https://vizhub.healthdata.org/burden-of-proof/)
Risk of bias in studies	18	Present assessments of risk of bias for each included study.	Supplemental Table 6
Results of individual studies	19	For all outcomes, present, for each study: (a) summary statistics for each group (where appropriate) and (b) an effect estimate and its precision (e.g. confidence/credible interval), ideally using structured tables or plots.	Supplemental Table 7
Results of syntheses	20a	For each synthesis, briefly summarise the characteristics and risk of bias among contributing studies.	First paragraph and last sentence of each risk-outcome pair results section
	20b	Present results of all statistical syntheses conducted. If meta-analysis was done, present for each the summary estimate and its precision (e.g. confidence/credible interval) and measures of statistical heterogeneity. If comparing groups, describe the direction of the effect.	Second paragraph of each risk-outcome pair results section + section titled “minimum risk level of vegetable intake;” Figures 1–5
	20c	Present results of all investigations of possible causes of heterogeneity among study results.	All uncertainty intervals presented everywhere in the manuscript and appendices reflect between-study heterogeneity (unless specified otherwise); BPRFs, ROSs, and star-ratings for each risk-outcome pair also reflect between-study heterogeneity
	20d	Present results of all sensitivity analyses conducted to assess the robustness of the synthesized results.	Supplemental Figures 1-6
Reporting biases	21	Present assessments of risk of bias due to missing results (arising from reporting biases) for each synthesis assessed.	Last sentence of each risk-outcome pair results section; funnel plots (figures 1C–5C)
Certainty of evidence	22	Present assessments of certainty (or confidence) in the body of evidence for each outcome assessed.	All estimates are presented with 95% uncertainty intervals. UI values are given alongside all mean estimates in the Results and Discussion sections; all risk curve figures (Figures 1–5) include shading to depict UI curves (both with and without between-study heterogeneity)

**DISCUSSION**			
Discussion	23a	Provide a general interpretation of the results in the context of other evidence.	Discussion paragraphs 2, 6–7
	23b	Discuss any limitations of the evidence included in the review.	Discussion paragraph 5
	23c	Discuss any limitations of the review processes used.	Discussion paragraph 5
	23d	Discuss implications of the results for practice, policy, and future research.	Discussion paragraphs 6–8
**OTHER INFORMATION**			
Registration and protocol	24a	Provide registration information for the review, including register name and registration number, or state that the review was not registered.	This systematic review was not registered, as stated in paragraph 3 of the methods overview
	24b	Indicate where the review protocol can be accessed, or state that a protocol was not prepared.	This systematic review was not registered, as stated in paragraph 3 of the methods overview
	24c	Describe and explain any amendments to information provided at registration or in the protocol.	This systematic review was not registered, as stated in paragraph 3 of the methods overview
Support	25	Describe sources of financial or non-financial support for the review, and the role of the funders or sponsors in the review.	"Acknowledgments" section of the manuscript
Competing interests	26	Declare any competing interests of review authors.	"Competing interests" section of the manuscript
Availability of data, code and other materials	27	Report which of the following are publicly available and where they can be found: template data collection forms; data extracted from included studies; data used for all analyses; analytic code; any other materials used in the review.	"Data availability" and "code availability" sections in the manuscript; data collection form template: Supplemental Table 5

Supplementary Table 2. PRISMA 2020 abstract checklist

Section and Topic	Item #	Checklist item	Reported (Yes/No)
**TITLE**			
Title	1	Identify the report as a systematic review.	Per the journal's request, the title does not include "systematic review". It is, however, in the title of the methods section, "conducting systematic reviews"
**BACKGROUND**			
Objectives	2	Provide an explicit statement of the main objective(s) or question(s) the review addresses.	Yes
**METHODS**			
Eligibility criteria	3	Specify the inclusion and exclusion criteria for the review.	Not in abstract, just main text (given word count limitations by the journal)
Information sources	4	Specify the information sources (e.g. databases, registers) used to identify studies and the date when each was last searched.	Not in abstract, just main text (given word count limitations by the journal)
Risk of bias	5	Specify the methods used to assess risk of bias in the included studies.	Not in abstract, just main text (given word count limitations by the journal)
Synthesis of results	6	Specify the methods used to present and synthesise results.	Yes
**RESULTS**			
Included studies	7	Give the total number of included studies and participants and summarise relevant characteristics of studies.	Not in abstract, just main text (given word count limitations by the journal)
Synthesis of results	8	Present results for main outcomes, preferably indicating the number of included studies and participants for each. If meta-analysis was done, report the summary estimate and confidence/credible interval. If comparing groups, indicate the direction of the effect (i.e. which group is favoured).	Yes, though number of included studies and participants only reported in the main text
**DISCUSSION**			
Limitations of evidence	9	Provide a brief summary of the limitations of the evidence included in the review (e.g. study risk of bias, inconsistency and imprecision).	Not in abstract, just main text (given word count limitations by the journal)
Interpretation	10	Provide a general interpretation of the results and important implications.	Yes
**OTHER**			
Funding	11	Specify the primary source of funding for the review.	Not in abstract, just main text (given word count limitations by the journal)
Registration	12	Provide the register name and registration number.	No

Section 3: Study characteristics

In Supplementary Table 3 we provide characteristics for each of the studies identified and used in this meta-analysis.

Supplementary Table 3. Studies characteristics

Author	Year	Study name	Population	Location	Study design	Sex	Follow-up	Age start	Age end	Exposure assessment	Endpoint	Disease ascertainment	Events	Sample size	Outcomes
Barouti	2022	Stockholm Diabetes Prevention Program	Residents aged 35-56 years from five municipalities in Stockholm County.	Sweden	Prospective cohort	Male	20	35	56	FFQ	Incidence	Biomarker	531	2646	Diabetes mellitus
Barouti	2022	Stockholm Diabetes Prevention Program	residents aged 35-56 years from five municipalities in Stockholm County	Sweden	Prospective cohort	Female	20	35	56	FFQ	Incidence	Biomarker	493	4315	Diabetes mellitus
Bazzano	2008	Nurses' Health Study	US female registered nurses	United States	Prospective cohort	Female	18	30	55	FFQ	Incidence	Physician diagnosis	4529	71346	Diabetes mellitus
Chen	2018	Singapore Chinese Health Study	Chinese permanent residents of government-built housing estates in Singapore	Singapore	Prospective cohort	Both	10.9	45	74	FFQ	Incidence	Physician diagnosis	5207	45411	Diabetes mellitus
Cooper	2012	EPIC-InterAct study	Sampled participants from 8 countries: France, Germany, Netherlands, UK, Italy, Sweden, Denmark, and Spain	Denmark, France, Germany, Italy, the Netherlands, Spain, Sweden, United Kingdom,	Case-cohort	Both	11	34.5	70.1	24-hr recall	Incidence	Administrative medical records or disease registries	10821	14800	Diabetes mellitus
Hodge	2004	Melbourne Collaborative Cohort study	Migrants from Italy and from Greece or Macedonia in Melbourne, Australia	Australia	Prospective cohort	Both	4	40	69	FFQ	Incidence	Self-report	365	31641	Diabetes mellitus
Kurotani	2013	Japan Public Health Center-based study	Residents aged 40-59 years in five Japanese public health center areas (Iwate, Akita, Nagano, Okinawa and Tokyo); the participants of cohort II included residents aged 40-69	Japan	Prospective cohort	Female	10	40	69	FFQ	Incidence	Self-report	366	27168	Diabetes mellitus

			years in six public health centre areas (Ibaraki, Niigata, Kochi, Nagasaki, Okinawa and Osaka)												
Kurotani	2013	Japan Public Health Center-based study	Residents aged 40-59 years in five Japanese public health Center areas (Iwate, Akita, Nagano, Okinawa and Tokyo); the participants of cohort II included residents aged 40-69 years in six public health centre areas (Ibaraki, Niigata, Kochi, Nagasaki, Okinawa and Osaka)	Japan	Prospective cohort	Male	10	40	69	FFQ	Incidence	Self-report	530	21269	Diabetes mellitus
Liu	2021	China Health and Nutrition Survey	CHNS, an ongoing multipurpose, longitudinal open cohort study initiated in 1989 in China and followed up every 2-4 years	China	Prospective cohort	Both	9	18	99	24-hr dietary recalls	Incidence	Self-report	1080	16117	Diabetes mellitus
Liu	2004	Womens Health study	Female health professionals who were free of heart disease, stroke, or cancer at baseline.	United States	Prospective cohort	Female	8.8	45	99	FFQ	Incidence	Self-report	1614	38018	Diabetes mellitus
Mamluk	2017	NIH-AARP Diet and Health study	US retired civilians	United States	Prospective cohort	Both	10.6	50	72	FFQ	Incidence	Self-report	25916	401909	Diabetes mellitus
Mamluk	2017	EPIC-Elderly study	Elderly individuals from Spain, Greece, Netherlands, and Sweden	Greece, The Netherlands, Spain, Sweden, United States	Prospective cohort	Both	11.8	50	72	FFQ	Incidence	Self-report	25916	20629	Diabetes mellitus
Meyer	2000	Iowa Womens Health study	Postmenopausal women	United states A	Prospective cohort	Female	6	55	69	FFQ	Incidence	Self-report	1141	35988	Diabetes mellitus
Montonen	2005	Finnish Mobile Clinic Health Examination survey	30 communities from different parts of Finland	Finland	Prospective cohort	Both	23	40	69	FFQ	Incidence	Administrative medical records or disease registries	383	4304	Diabetes mellitus

Olsson	2021	Malmö Diet and Cancer Study	men born between 1923 and 1950 and women born between 1923 and 1950 in Malmö, Sweden	Sweden	Prospective cohort	Male	18.4	46	73	FFQ	Incidence	Administrative medical records or disease registries	1936	10315	Diabetes mellitus
Olsson	2021	Malmö Diet and Cancer Study	men born between 1923 and 1950 and women born between 1923 and 1950 in Malmö, Sweden	Sweden	Prospective cohort	Female	18.4	41	73	FFQ	Incidence	Administrative medical records or disease registries	2110	16307	Diabetes mellitus
Qiao	2014	Women's Health Initiative study	US postmenopausal women	United States	Prospective cohort	Female	7.6	50	79	FFQ	Incidence	Self-report	10285	154493	Diabetes mellitus
Villegas	2008	Shanghai Women's Health Study	Middle-aged Chinese women from Shanghai	China	Prospective cohort	Female	4.6	40	70	FFQ	Incidence	Self-report	1608	64227	Diabetes mellitus
Ahmed	2020	Stockholm Public Health Cohort	Stockholm area-stratified random sample of adults aged 18-84 years	Sweden	Prospective cohort	Male	4	25	84	dietary recall	Incidence	Self-report	167	14718	Diabetes mellitus
Ahmed	2020	Stockholm Public Health Cohort	Stockholm area-stratified random sample of adults aged 18-84 years	Sweden	Prospective cohort	Female	4	25	84	dietary recall	Incidence	Self-report	152	20589	Diabetes mellitus
Rayner	2020	Australian Longitudinal Study on Women's Health	Nationally representative survey of Australian women	Australia	Prospective cohort	Female	20	18	75	FFQ	Incidence	Self-report	959	9689	Diabetes mellitus
Freedman	2007	NIH-AARP Diet and Health study	AARP members residing in 6 US states (California, Florida, Louisiana, New Jersey, North Carolina and Pennsylvania) and 2 metropolitan areas (Atlanta, Georgia and Detroit, Michigan).	United States	Prospective cohort	Both	5	61.6	61.6	FFQ	Incidence	Administrative medical records or disease registries	103	98160	Esophageal cancer
González	2006	EPIC-EURGAST	Residents mostly aged 35-70 years from a given geographical area, town, or province. Exceptions were the French cohort based	Denmark, France, Germany, Greece, Italy, the Netherlands, Norway,	Prospective cohort	Both	6.5	35	70	FFQ	Incidence & Mortality	Administrative medical records or disease registries	65	481518	Esophageal cancer

			on members of the health insurance of school employees, the Utrecht cohort and the Florence cohort based on women attending breast cancer screening, part of the Italian and Spanish cohort based on blood donors and most of the Oxford cohort based on vegetarian volunteers	Spain, Sweden and the United Kingdom											
Sheikh	2019	Golestan Cohort Study	Residents aged 40-75 years from Golestan province in north eastern Iran	Iran	Prospective cohort	Both	10	40	75	FFQ and 24-hr dietary recall.	Incidence & Mortality	Self-report	317	49155	Esophageal cancer
Steevens	2011	Netherlands Cohort Study on Diet and Cancer	Randomly sampled from Dutch municipal registries	The Netherlands	Prospective cohort	Both	16.3	55	69	FFQ	Incidence	Administrative medical records or disease registries	101	120852	Esophageal cancer
Tran	2005	Linxian General Population Trial	Individuals with no history of cancer or debilitating disease at baseline from the general population of Linxian.	China	Prospective cohort	Both	15	40	69	FFQ	Incidence	Physician diagnosis	1958	29584	Esophageal cancer
Yamaji	2008	Japan Public Health Center-based Prospective Study	All male registered Japanese inhabitants in 11 public health center (PHC) areas, aged 40-59 years in Cohort I and 40-69 years in Cohort II. Study subjects were identified using population registries maintained by the local municipalities. Excluded 2 public health center areas (Toyko and Osaka)	Japan	Prospective cohort	Male	7.7	40	59	FFQ	Incidence & Mortality	Administrative medical records or disease registries	116	38790	Esophageal cancer
Larsson	2009	ATBC study	male smokers residing in southwestern Finland	Finland	Prospective cohort	Male	13.6	50	69	FFQ	Incidence	Administrative medical records or disease registries	383	26556	Hemorrhagic stroke

Larsson	2013		Men and women from central Sweden	Sweden	Prospective cohort	Both	10.2	45	83	FFQ	Incidence	Administrative medical records or disease registries	583	74961	Hemorrhagic stroke
Mizrahi	2009	Finnish Mobile Clinic Health Examination Survey	40-74 years of age and free of cardiovascular diseases at baseline.	Finland	Prospective cohort	Male	24	40	74	FFQ	Incidence	Administrative medical records or disease registries	65	2051	Hemorrhagic stroke
Nagura	2009	Japan Collaborative Cohort Study	aged 40–79 years living in forty-five communities across Japan participated in municipal health screening examinations	Japan	Prospective cohort	Both	13	40	79	FFQ	Mortality	Administrative medical records or disease registries	393	59485	Hemorrhagic stroke
Pham	2007	Miyako Study	Four selected districts in Fukoka Prefecture, Japan	Japan	Prospective cohort	Both	13.8	40	78.6	Self-administered questionnaire survey	Mortality	Death certificates	47	9651	Hemorrhagic stroke
Yokoyama	2000	The Shibata Study	Residents of Akadani-Ijimino district of Shibata located in the northern part of Niigata Prefecture, Japan.	Japan	Prospective cohort	Female	20	40	99	FFQ	Incidence	Self-report	36	1251	Hemorrhagic stroke
Yokoyama	2000	The Shibata Study	Residents of Akadani-Ijimino district of Shibata located in the northern part of Niigata Prefecture, Japan.	Japan	Prospective cohort	Male	20	40	99	FFQ	Incidence	Self-report	18	870	Hemorrhagic stroke
Zhang	2011		Participants of cross-sectional population surveys performed in 6 geographic areas of Finland in 1982, 1987, 1992, 1997, and 2002	Finland	Prospective cohort	Both	13.7	25	64	FFQ	Incidence & Mortality	Administrative medical records or disease registries	311	36686	Hemorrhagic stroke
Bhupathiraju	2013	Nurses' Health Study	US female registered nurses	United States	Prospective cohort	Female	24	30	55	FFQ	Incidence	Administrative medical records or disease registries	2582	113276	Ischemic heart disease
Bhupathiraju	2013	Health Professionals Follow-Up study	US male health professionals	United States	Prospective cohort	Male	22	40	75	FFQ	Incidence	Administrative medical records or disease registries	3607	113276	Ischemic heart disease

Dauchet	2010	PRIME study	Four WHO-MONICA centers in Belfast (UK), Lille (Northern France), Strasbourg (Eastern France) and Toulouse (Southwestern France).	France and Northern Ireland	Prospective cohort	Male	10	50	59	FFQ	Incidence & Mortality	Administrative medical records or disease registries	367	2297	Ischemic heart disease
Dauchet	2010	PRIME study	Four WHO-MONICA centers in Belfast (UK), Lille (Northern France), Strasbourg (Eastern France) and Toulouse (Southwestern France).	France and Northern Ireland	Prospective cohort	Male	10	50	59	FFQ	Incidence & Mortality	Administrative medical records or disease registries	367	2410	Ischemic heart disease
Dauchet	2010	PRIME study	Four WHO-MONICA centers in Belfast (UK), Lille (Northern France), Strasbourg (Eastern France) and Toulouse (Southwestern France).	France and Northern Ireland	Prospective cohort	Male	10	50	59	FFQ	Incidence & Mortality	Administrative medical records or disease registries	367	3353	Ischemic heart disease
Hansen	2010	Danish Diet, Cancer, and Health Study	All men and women aged 50 to 64 years, born in Denmark, living in the greater areas of Aarhus or Copenhagen, and with no previous cancer diagnosis in the Danish Cancer Registry were invited	Denmark	Prospective cohort	Male	7.7	50	64	FFQ	Incidence & Mortality	Administrative medical records or disease registries	820	25065	Ischemic heart disease
Hansen	2010	Danish Diet, Cancer, and Health Study	All men and women aged 50 to 64 years, born in Denmark, living in the greater areas of Aarhus or Copenhagen, and with no previous cancer diagnosis in the Danish Cancer Registry were invited	Denmark	Prospective cohort	Female	7.7	50	64	FFQ	Incidence & Mortality	Administrative medical records or disease registries	255	28318	Ischemic heart disease

Knekt	1994	Finnish Mobile Health Clinic	Adult men and women from rural, urban, and industrial communities in Finland	Finland	Prospective cohort	Female	14	30	69	FFQ	Mortality	Death certificates	244	2385	Ischemic heart disease
Knekt	1994	Finnish Mobile Health Clinic	Adult men and women from rural, urban, and industrial communities in Finland	Finland	Prospective cohort	Male	14	30	69	FFQ	Mortality	Death certificates	244	2748	Ischemic heart disease
Kobylecki	2015	CGPS and the Copenhagen City Heart Study	Iindividuals aged 20–100 y were invited randomly from the Danish Civil Registration System t	Denmark	Prospective cohort	Both	21	20	100	FFQ	Incidence	Administrative medical records or disease registries	2823	97203	Ischemic heart disease
Liu	2000	Womens' Health Study	Female health professionals who were without heart disease, stroke, or cancer (other than nonmelanoma skin cancer) at baseline.	United States	Prospective cohort	Female	5	40	68	FFQ	Incidence & Mortality	Self-report	126	39876	Ischemic heart disease
Miller	2017	PURE	613 communities in 18 low-income, middle-income, and high-income countries (HIC) in seven geographical regions: North America and Europe, South America, the Middle East, south Asia, China, southeast Asia, and Africa.	United Arab Emirates, Canada, Sweden, Poland, Argentina, Chile, Malasia, Turkey, Iran, Occupied Palestinian territory, Brazil, South Africa, Columbia, China, India, Pakistan, Bangladesh, Zimbabwe	Prospective cohort	Both	7.4	35	70	FFQ	Incidence	Physician diagnosis	2143	135335	Ischemic heart disease
Perez-Cornago	2021	European Prospective Investigation into Cancer and Nutrition	Men and women recruited through 23 centers in 10 European countries (Denmark, France, Germany, Greece, Italy, The	Denmark, France, Germany, Greece, Italy, The Netherlands, Norway,	Prospective cohort	Female	12.6	35	70	FFQ	Incidence & Mortality	Administrative medical records or disease registries	8504	490311	Ischemic heart disease

			Netherlands, Norway, Spain, Sweden and the UK)	Spain, Sweden and the UK											
Pietinen	1996	Alpha-Tocopherol, Beta-Carotene Cancer Prevention Study	Male smokers recruited from 14 geographic areas in southwestern Finland	Finland	Prospective cohort	Male	6.1	50	69	FFQ	Incidence & Mortality	Administrative medical records or disease registries	1399	29133	Ischemic heart disease
Rebello	2014	Singapore Chinese Health Study	Singapore citizens or permanent residents aged between 45-74 y who were residing at public housing estates	Singapore	Prospective cohort	Female	15	45	74	FFQ	Mortality	Administrative medical records or disease registries	638	29968	Ischemic heart disease
Rebello	2014	Singapore Chinese Health Study	Singapore citizens or permanent residents aged between 45-74 y who were residing at public housing estates	Singapore	Prospective cohort	Male	15	45	74	FFQ	Mortality	Administrative medical records or disease registries	1022	23501	Ischemic heart disease
Sharma	2014	MEC study	Large representative samples of five ethnic groups in the United States; Caucasian, African American, Native Hawaiian, Japanese American, and Latino.	United States	Prospective cohort	Male	8	45	75	FFQ	Mortality	Administrative medical records or disease registries	1140	72866	Ischemic heart disease
Sharma	2014	MEC study	Large representative samples of five ethnic groups in the United States; Caucasian, African American, Native Hawaiian, Japanese American, and Latino.	United States	Prospective cohort	Female	8	45	75	FFQ	Mortality	Administrative medical records or disease registries	811	91940	Ischemic heart disease
Sonestedt	2015	Malmö Diet and Cancer Study	All men born between 1923 and 1945 and women born between 1923 and 1950 that live in Malmö were invited via personal letters and advertisements in the local newspaper and	Sweden	Prospective cohort	Both	14	44	74	7-day diet history	Incidence & Mortality	Administrative medical records or disease registries	2921	26445	Ischemic heart disease

			public places to participate in the study												
Stefler	2016	HAPIEE study	Middle-aged men and women, randomly selected from population/electoral registers in Krakow (Poland), Novosibirsk (Russia) and six cities of the Czech Republic	Czech Republic, Poland and Russia.	Prospective cohort	Both	7.1	43	71	FFQ	Mortality	Administrative medical records or disease registries	226	19333	Ischemic heart disease
Tognon	2014	MONICA	Randomly selected from the Central Person Register of citizens born in 1922, 1932, 1942 and 1952 living in eleven municipalities in the Copenhagen County.	Denmark	Prospective cohort	Both	14	30	85	7d food record	Incidence & Mortality	Administrative medical records or disease registries	161	1849	Ischemic heart disease
Yoshizaki	2020	Japan Public Health Center-Based Prospective Study	Residents of the Okinawa Prefecture (two PHC areas: Chubu from Cohort I and Miyako from Cohort II)	Japan	Prospective cohort	Male	13.2	40	74	FFQ	Incidence & Mortality	Administrative medical records or disease registries	147	7726	Ischemic heart disease
Yoshizaki	2020	Japan Public Health Center-Based Prospective Study	Residents of the Okinawa Prefecture (two PHC areas: Chubu from Cohort I and Miyako from Cohort II)	Japan	Prospective cohort	Female	13.2	40	74	FFQ	Incidence & Mortality	Administrative medical records or disease registries	32	8772	Ischemic heart disease
Yu	2014	Shanghai Women's Health Study (SWHS)	Participants from the Shanghai Women’s Health Study (SWHS) and the Shanghai Men’s Health Study (SMHS)	China	Prospective cohort	Female	9.8	40	70	FFQ	Incidence	Physician diagnosis	148	67211	Ischemic heart disease
Yu	2014	Shanghai Men's Health Study (SMHS)	Participants from the Shanghai Women’s Health Study (SWHS) and the Shanghai Men’s Health Study (SMHS)	China	Prospective cohort	Male	5.4	40	74	FFQ	Incidence	Physician diagnosis	217	55474	Ischemic heart disease

Zhang	2021	UK Biobank study	CVD-free participants aged 40-69y	United Kingdom	Prospective cohort	Female	11.2	40	69	FFQ	Incidence	Administrative medical records or disease registries	11161	462155	Ischemic heart disease
Johnsen	2003	Diet, Cancer, and Health study	Persons living in the Copenhagen and Aarhus areas	Denmark	Prospective cohort	Both	3.1	50	64	FFQ	Incidence & Mortality	Administrative medical records or disease registries	266	57053	Ischemic stroke
Joshipura	1999	Nurses' Health Study	US female registered nurses	United States	Prospective cohort	Female	14	34	59	FFQ	Incidence & Mortality	Administrative medical records or disease registries	366	75596	Ischemic stroke
Joshipura	1999	Health Professionals Follow-Up study	US male health professionals	United States	Prospective cohort	Male	8	40	75	FFQ	Incidence & Mortality	Administrative medical records or disease registries	204	38683	Ischemic stroke
Larsson	2009	ATBC study	Male smokers residing in southwestern Finland	Finland	Prospective cohort	Male	13.6	50	69	FFQ	Incidence	Administrative medical records or disease registries	2702	26556	Ischemic stroke
Larsson	2013		Men and women from central Sweden	Sweden	Prospective cohort	Both	10.2	45	83	FFQ	Incidence	Administrative medical records or disease registries	3159	74961	Ischemic stroke
Mizrahi	2009	Finnish Mobile Clinic Health Examination Survey	40-74 years of age and free of cardiovascular diseases at baseline.	Finland	Prospective cohort	Male	24	40	74	FFQ	Incidence	Administrative medical records or disease registries	342	2051	Ischemic stroke
Nagura	2009	Japan Collaborative Cohort Study	aged 40–79 years living in forty-five communities across Japan participated in municipal health screening examinations	Japan	Prospective cohort	Both	13	40	79	FFQ	Mortality	Administrative medical records or disease registries	362	59485	Ischemic stroke
Pham	2007	Miyako Study	Four selected districts in Fukoka Prefecture, Japan	Japan	Prospective cohort	Both	13.8	40	78.6	Self-administered questionnaire survey	Mortality	Death certificates	109	9651	Ischemic stroke
Steffen	2003	ARIC study	African American and white, men and women residents from Forsyth County, NC; Jackson, MS; selected suburbs of	United States	Prospective cohort	Both	11	45	64	FFQ	Incidence	Administrative medical records or disease registries	214	11940	Ischemic stroke

			Minneapolis; and Washington County, MD												
Tong	2020	EPIC	Men and women from 22 Centers in nine European countries (Denmark, Germany, Greece, Italy, the Netherlands, Norway, Spain, Sweden, and the UK)	Denmark, Germany, Greece, Italy, the Netherlands, Norway, Spain, Sweden, and the UK	Prospective cohort	Both	12.7	35	70	FFQ	Incidence & Mortality	Administrative medical records or disease registries	4281	418329	Ischemic stroke
Yokoyama	2000	The Shibata Study	Residents of Akadani-Ijimino district of Shibata located in the northern part of Niigata Prefecture, Japan.	Japan	Prospective cohort	Male	20	40	99	FFQ	Incidence	Self-report	58	870	Ischemic stroke
Yokoyama	2000	The Shibata Study	Residents of Akadani-Ijimino district of Shibata located in the northern part of Niigata Prefecture, Japan.	Japan	Prospective cohort	Female	20	40	99	FFQ	Incidence	Self-report	51	1251	Ischemic stroke
Yoshizaki	2020	JPHC	Adults from Okinawa Prefecture (two PHC areas: Chubu from Cohort I and Miyako from Cohort II)	Japan	Prospective cohort	Male	13.2	45	74	FFQ	Incidence & Mortality	Administrative medical records or disease registries	298	7726	Ischemic stroke
Yoshizaki	2020	JPHC	Adults from Okinawa Prefecture (two PHC areas: Chubu from Cohort I and Miyako from Cohort II)	Japan	Prospective cohort	Female	13.2	45	74	FFQ	Incidence & Mortality	Administrative medical records or disease registries	168	8772	Ischemic stroke
Zhang	2011		Participants of cross-sectional population surveys performed in 6 geographic areas of Finland in 1982, 1987, 1992, 1997, and 2002	Finland	Prospective cohort	Both	13.7	25	64	FFQ	Incidence & Mortality	Administrative medical records or disease registries	1167	36686	Ischemic stroke

Tong	2020	EPIC	Men and women from 22 centres in nine European countries (Denmark, Germany, Greece, Italy, the Netherlands, Norway, Spain, Sweden, and the UK)	Denmark, Germany, Greece, Italy, the Netherlands, Norway, Spain, Sweden, and the UK	Prospective cohort	Both	12.7	35	70	FFQ	Incidence & Mortality	Administrative medical records or disease registries	1430	418329	Hemorrhagic stroke
Yoshizaki	2020	JPHC	Adults from Okinawa Prefecture (two PHC areas: Chubu from Cohort I and Miyako from Cohort II)	Japan	Prospective cohort	Male	13.2	45	74	FFQ	Incidence & Mortality	Administrative medical records or disease registries	159	7726	Hemorrhagic stroke
Yoshizaki	2020	JPHC	Adults from Okinawa Prefecture (two PHC areas: Chubu from Cohort I and Miyako from Cohort II)	Japan	Prospective cohort	Female	13.2	45	74	FFQ	Incidence & Mortality	Administrative medical records or disease registries	132	8772	Hemorrhagic stroke

Section 4. GATHER checklists

Supplementary Table 4. GATHER checklist

Item #	Checklist item	Reported on page #
Objectives and funding		
1	Define the indicator(s), populations (including age, sex, and geographic entities), and time period(s) for which estimates were made.	Main text methods overview, paragraph 2
2	List the funding sources for the work.	Main text acknowledgement section
Data Inputs		
For all data inputs from multiple sources that are synthesized as part of the study:		
3	Describe how the data were identified and how the data were accessed.	Main text methods section "conducting systematic reviews"
4	Specify the inclusion and exclusion criteria. Identify all ad-hoc exclusions.	Main text methods section "conducting systematic reviews"; reasons for exclusion and number of studies excluded also provided in PRISMA flow diagram (Extended Data Figures 1–4)
5	Provide information on all included data sources and their main characteristics. For each data source used, report reference information or contact name/institution, population represented, data collection method, year(s) of data collection, sex and age range, diagnostic criteria or measurement method, and sample size, as relevant.	SI section 3, Supplemental Table 3 ("study characteristics"); citations also available for download from the online viz tools: https://vizhub.healthdata.org/burden-of-proof/
6	Identify and describe any categories of input data that have potentially important biases (e.g., based on characteristics listed in item 5).	Main text methods section "testing and adjusting for biases across study designs and characteristics" and "evaluating potential for publication or reporting bias"
For data inputs that contribute to the analysis but were not synthesized as part of the study:		
7	Describe and give sources for any other data inputs.	N/A
For all data inputs:		
8	Provide all data inputs in a file format from which data can be efficiently extracted (e.g., a spreadsheet rather than a PDF), including all relevant meta-data listed in item 5. For any data inputs that cannot be shared because of ethical or legal reasons, such as third-party ownership, provide a contact name or the name of the institution that retains the right to the data.	As stated in the Data Availability Statement, data inputs in excel format available for download from the online viz tools: https://vizhub.healthdata.org/burden-of-proof/
Data analysis		
9	Provide a conceptual overview of the data analysis method. A diagram may be helpful.	Main text methods overview, paragraph 1
10	Provide a detailed description of all steps of the analysis, including mathematical formulae. This description should cover, as relevant, data cleaning, data pre-processing, data adjustments and weighting of data sources, and mathematical or statistical model(s).	Main text methods
11	Describe how candidate models were evaluated and how the final model(s) were selected.	Main text methods "estimating the shape of the exposure-relative risk relationship" paragraph 2
12	Provide the results of an evaluation of model performance, if done, as well as the results of any relevant sensitivity analysis.	Main text methods "sensitivity analyses" section; Supplemental Figures 1-6
13	Describe methods for calculating uncertainty of the estimates. State which sources of uncertainty were, and were not, accounted for in the uncertainty analysis.	Main text methods "evaluating between-study heterogeneity, uncertainty, and small numbers of studies"
14	State how analytic or statistical source code used to generate estimates can be accessed.	Code availability statement in the main text
Results and Discussion		
15	Provide published estimates in a file format from which data can be efficiently extracted.	Available at: https://vizhub.healthdata.org/burden-of-proof/
16	Report a quantitative measure of the uncertainty of the estimates (e.g. uncertainty intervals).	UIs given for all findings, including in the text, figures, and tables in the main text and SI; online viz tools (see information above)
17	Interpret results in light of existing evidence. If updating a previous set of estimates, describe the reasons for changes in estimates.	Main text discussion paragraphs 2, 6, & 7
18	Discuss limitations of the estimates. Include a discussion of any modelling assumptions or data limitations that affect interpretation of the estimates.	Main text discussion paragraph 5

Section 5: Causal criteria

For reports that met the inclusion criteria, data were extracted for the variables listed in Supplemental Table 5.

Supplementary Table 5. Causal criteria extraction template

Category	Variable	Definition
Source	seq	
	underlying_nid	Underlying NID: Enter the underlying NID of the study (if applicable). Always talk to a data indexer if you don't know if an underlying NID is needed. They may be used for meta-analyses, certain database sources, and in some other specific cases.
	nid	Found in GHDx, created through the epi form, or created by Data Indexer
	field_citation_value	IHME Zotero format or if source has NID, citation info from GHDx
	file_path	optional; full file path of article; Only needed if source doesn't have NID, to facilitate NID creation.
R-O pair	risk	Risk: Select the risk factor, if not listed here, contact the causal criteria team
	risk_mapping	the relationship between study definition of risk and GBD definition of risk for a particular effect size
	outcome	Outcome: Select the outcome.
	outcome_mapping	the relationship between study definition of outcome and GBD definition of outcomefor a particular effect size
Location	location_name	location name (from locations tab). Do a fast double-click in this field to get the drop-down menu, then start typing the location_name. For location_names with special characters, you may need to use the scroll bar.
	location_id	autopopulated from location_name
	rep_geography	Were the study partcipants representative of the geography? 1=yes, 0=no
	rep_selection_criteria	If rep_geography is 0, please specify the selection criteria of the study that is used in the analysis
	rep_prevalent_disease	Is the study aiming to evaluate the risk or mortality of people who have already developed the outcome? 1=yes 0=no (i.e. yes if for SBP-IHD paper, all partcipants have IHD at baseline and the paper is looking at mortality due to SBP, no if for SBP-IHD paper the participants have other prevalent diseases)
Study Population	year_start_study	year the study was started. If not specified, leave blank
	year_end_study	year the study was finished (including most recent follow up). If not specified, leave blank
	age_start	ages from 1 and above must be entered as an integer. Ages <1 can be entered as decimal values, e.g., 3 days = 3/365.
	age_end	ages from 1 and above must be entered as an integer. Ages <1 can be entered as decimal values, e.g., 3 days = 3/365.
	age_mean	Mean age
	age_sd	SD of age
	age_issue	0 = no issue flagged; 1 = issue flagged for modeler; always include explanatory notes the note_SR column
	percent_male	what percent of the population is male (0-1), if pop is all female then it would be 0
	sex_issue	sex_issue
Study Design	design	Study design: Specify the design of the study
	study_name	Study Name: Enter the name of the study (e.g., Nurses' Health Study), if provided. Do not enter the title of the article.
Exposure	exp_assess_level	Level of exposure assessment: The exposure was assessed…
	exp_instrument	Exposure assessment instrument: Specify the name of the exposure assessment instrument. For self-reported exposures, please specify the name of the questionnaire e.g., International Physical Activity Questionnaire (IPAQ). If more than one instrument specify all
	exp_assess_period	What was the frequency of exposure assessment?
	exp_assess_num	if multiple, specify the number of times that exposure was assessed (excluding baseline)
	exp_method_1	Please specify the method of exposure assessment. If there are more than 1, please add in the next columns labeled "exp_method_2".
	exp_method_2	Please specify the method of exposure assessment. If there are more than 2, please add in the next columns labeled "exp_method_3".
	exp_method_3	Please specify the method of exposure assessment.

	exp_recall_period	This field describes the unit of exposure recall used in data collection ONLY for self-report. Select the correct option from the drop-down menu. If the unit is days, weeks, months, or years, please enter the number in exp_recall_period_value (next column). If the unit is 'lifetime', nothing needs to be entered in exp_recall_period_value. For example, if the study said the recall period was 4 weeks, enter 4 in exp_recall_period_value, and 'weeks' in the field exp_recall_period. If 'other' is selected, please describe in exp_recall_period_other
	exp_recall_period_value	If you entered days, weeks, months, or years in the field 'exp_recall_period', please enter the corresponding integer in this field. For example, if the study said the recall period was 4 weeks, enter 4 in exp_recall_period_value, and 'weeks' in the field exp_recall_period.
	exp_recall_period_other	If 'other' was selected in exp_recall_period, please describe the exposure recall period that the study specified (e.g., recall of exposure from 12 to 18 years).
	exp_type	Which form of the exposure was included in relative risk estimation analysis?
Outcome	outcome_def	Outcome definition: Provide a brief description of the outcome as reported in the study.
	outcome_type	Outcome type: please specify if the outcome definition included incidence of or mortality from a disease endpoint
	outcome_assess_1	Method of outcome assessment: Specify the method of assessment of the study outcome. If more than 1 are appropriate, enter additional methods in the next column labeled "outcome_assess_2"
	outcome_assess_2	Method of outcome assessment: Specify the method of assessment of the study outcome. If more than 2 are appropriate, enter additional methods in the next column labeled "outcome_assess_3"
	outcome_assess_3	Method of outcome assessment: Specify the method of assessment of the study outcome.
Follow up	duration_fup_measure	Type of follow up measure (i.e. mean, median, max, min)
	duration_fup_units	Units of follow up duration
	value_of_duration_fup	Enter the length of participant follow-up.
Confounders	confounders_age	if controlled for in the relative risk estimation analysis, mark 1 for yes. Mark 0 for no
	confounders_sex	if controlled for in the relative risk estimation analysis, mark 1 for yes. Mark 0 for no
	confounders_education	if controlled for in the relative risk estimation analysis, mark 1 for yes. Mark 0 for no
	confounders_income	if controlled for in the relative risk estimation analysis, mark 1 for yes. Mark 0 for no
	confounders_smoking	if controlled for in the relative risk estimation analysis, mark 1 for yes. Mark 0 for no
	confounders_alcohol_use	if controlled for in the relative risk estimation analysis, mark 1 for yes. Mark 0 for no
	confounders_physical_activity	if controlled for in the relative risk estimation analysis, mark 1 for yes. Mark 0 for no
	confounders_dietary_components	if controlled for in the relative risk estimation analysis, mark 1 for yes. Mark 0 for no
	confounders_bmi	if controlled for in the relative risk estimation analysis, mark 1 for yes. Mark 0 for no
	confounders_hypertension	if controlled for in the relative risk estimation analysis, mark 1 for yes. Mark 0 for no
	confounders_diabetes	if controlled for in the relative risk estimation analysis, mark 1 for yes. Mark 0 for no
	confounders_hypercholesterolemia	if controlled for in the relative risk estimation analysis, mark 1 for yes. Mark 0 for no
	confounders_other	For other confounders that not listed, list here
Effect Size	page_num_effect_size	Page number (where you found effect_size) from literature, or survey question where you found effect size; Use page number(s) of article, not page # of pdf
	effect_size_measure	Effect size measure: Specify the measure of effect size
	effect_size	Effect size estimate: Provide the effect size estimate
	lower	Provide the lower limit of the confidence interval. Enter on a "per 1" basis. (If the CI is reported as a percent, you must convert to a decimal.) These 3 fields must all be filled in if any of them are filled in: lower, upper, uncertainty_type_value.

	upper	Provide the upper limit of the confidence interval. Enter on a "per 1" basis. (If the CI is reported as a percent, you must convert to a decimal.) These 3 fields must all be filled in if any of them are filled in: lower, upper, uncertainty_type_value.
	CI_uncertainty_type_value	This field is required if 'lower' & 'upper' are entered. This column represents the confidence level which is reported at (Eg. 95, 90, 99). These 3 fields must all be filled in if any of them are filled in: lower, upper, uncertainty_type_value.
	nonCI_uncertainty_value	Numerical value of the nonCI_uncertainty_type entered in that column. For example, if SD=5.3, you'd put 5.3 in this column, and choose SD from the drop down menu in nonCI_uncertainty_type.
	nonCI_uncertainty_type	Enter SE or SD if appropriate. For example, if SD=5.3, you'd put 5.3 in nonCI_uncertainty_value, and choose SD from the drop down menu in this column (nonCI_uncertainty_type).
	uncertainty_issue	Mark with a 1 if no uncertainty is reported, if some sort of uncertainty is reported, mark 0
	subgroup_analysis	1 if RR is from main analysis (all participants), 0 if sub-analysis (only males, or among a specific age group, etc.)
	subgroup_analysis_free_text	if a sub-analysis, describe it (i.e. age, sex, etc.)
	effect_size_multi_location	1 if the reported effect size is from a multi-country study and only one effect size has been reported for all locations, otherwise 0
	effect_size_multi_location_specify	which geography level is the RR for
	pooled_cohort	1 if the reported effect size is from a pooled analysis and only pooled effect size has been reported, otherwise 0
	dose_response	Does the study support a dose-response relationship between the exposure and the outcome? (1= yes, 0=no)
	dose_response_detail	If "1" was specified in the dose_response field, please specify in this field the type of evidence supporting the dose-response relationship. For example, "statistically significant p value for linear trend".
Cohorts	cohort_person_years_exp	Please specify the person years of follow up in the exposed group
	cohort_person_years_unexp	Please specify the person years of follow up in the unexposed group
	cohort_person_years_total	Enter the total person-years of follow-up if person-years of follow up in exposed and unexposed not reported
	cohort_number_events_exp	Please specify the number of events in the exposed group
	cohort_number_events_unexp	Please specify the number of events in the unexposed group
	cohort_number_events_total	Enter the total number of events/cases if number of events in exposed and unexpoxed not reported
	cohort_sample_size_exp	Please specify the number of people in the exposed group if person-years of follow up in exposed not reported
	cohort_sample_size_unexp	Please specify the number of people in the unexposed group if person-years of follow up in unexposed not reported
	cohort_sample_size_total	Please specify the number of people included in the analysis if total person-years of follow up in not reported
	cohort_dropout_rate	Dropout rate: Specify the dropout rate (%) at the end of the study. Enter on a "per 1" basis. For example: 23% is entered as .23.
	cohort_dropout_assess	Specify how dropout rate was defined in the study.
	cohort_exposed_def	exposed group definition: Provide a brief description of the exposed group (i.e., the comparison group) as used in estimation of the relative risk (e.g., never smokers)
	cohort_exp_unit_rr	Exposure unit (for continuous risks): Specify the unit of exposure (e.g., grams/day).
	cohort_exp_level_rr	Exposure level in the exposed group (for continuous risks): Specify the mean/median level of exposure in the exposed group.
	cohort_unexp_def	unexposed group definition: Provide a brief description of the unexposed group (i.e., the comparison group) as used in estimation of the relative risk (e.g., never smokers)
	cohort_unexp_unit_rr	Exposure unit (for continuous risks): Specify the unit of exposure (e.g., grams/day) for the unexposed group
	cohort_unexp_level_rr	Exposure level in the unexposed group (for continuous risks): Specify the mean/median level of exposure in the unexposed group.
	cohort_exp_level_dr	Exposure level in for dose-repose RRs (for continuous risks): If the study reports dose-repose RR, please specify the level of exposure for the reported RR
Case-control	cc_community	Were the controls selected from the community? 1 = yes, 0=no
	cc_cases	Number of cases

	cc_control	Number of controls
	cc_exposed_def	Exposed group definition: Provide a brief description of the exposed group for which the the relative risk is reported (e.g., current smokers)
	cc_exp_unit_rr	Exposure unit (for continuous risks): Specify the unit of exposure (e.g., grams/day).
	cc_exp_level_rr	Exposure level in the exposed group (for continuous risks): Specify the mean/median level of exposure in the exposed group.
	cc_unexposed_def	Unexposed group definition: Provide a brief description of the unexposed group (i.e., the comparison group) as used in estimation of the relative risk (e.g., never smokers)
	cc_unexp_unit_rr	
	cc_unexp_level_rr	Exposure level in the unexposed group (for continuous risks): Specify the mean/median level of exposure in the unexposed group.
	cc_exp_level_dr	Exposure level in for dose-repose RRs (for continuous risks): If the study reports dose-repose RR, please specify the level of exposure for the reported RR
Trials	int_intervention_description	Intervention definition: Provide a brief description of the intervention as reported in the study.
	int_control_description	control definition: Provide a brief description of the control as reported in the study.
	int_intervention_multi_rf	Does this intervention simultaneously target more than one risk? (1=yes, 0=no)
	int_intervention_multi_rf_specify	Specify the risks that are targted by the interevention
	int_intervention_level	Level of intervention: The intervention was implemented …
	int_adhere_assess	Specify how adherence was defined in the study.
	int_adhere_rate_intervention	adherence rate in the intervention group; Enter on a "per 1" basis. For example: 23% is entered as .23.
	int_adhere_rate_control	adherence rate in the control group; Enter on a "per 1" basis. For example: 23% is entered as .23.
	int_dropout_rate_intervention	Dropout rate in the intervention group: Specify the dropout rate (%) at the end of the study. Enter on a "per 1" basis. For example: 23% is entered as .23.
	int_dropout_rate_control	Dropout rate in the control group: Specify the dropout rate (%) at the end of the study. Enter on a "per 1" basis. For example: 23% is entered as .23.
	int_dropout_assess	Specify how dropout rate was defined in the study.
	int_blinding	For interventional studies. Blinding: The trial was … (select 1)
	int_exp_unit	For trials, specify the unit of exposure (e.g., mmol/l)
	int_baseline_exp_int	For trials, specify the exposure level in the intervention group at baseline
	int_baseline_exp_comp	For trials, specify the exposure level in the comparison group at baseline
	int_fup_exp_int	For trials, specify the exposure level in the intervention group at the end of the follow-up time
	int_fup_exp_comp	For trials, specify the exposure level in the comparison group at the end of follow up time
	int_fup_exp_int_difference	For trials, please specify the difference of exposure level between baseline and follow up time for the intervention group
	int_fup_exp_comp_difference	For trials, please specify the difference of exposure level between baseline and follow up time for the comparison grouo
	int_person_years_int	Please specify the number of person years of follow up for the intrevention group
	int_person_years_comp	Please specify the number of person years of follow up in the comparison group
	int_number_events_int	For trials, specify the number of cases in the intervention group at the end of follow up
	int_number_events_comp	For trials, specify the number of cases in the control group at the end of follow up
	int_sample_size_int_group_baseline	For trials, specify the sample size in the intervention group at baseline
	int_sample_size_comparison_group_baseline	For trials, specify the sample size in the comparison group at baseline
	int_sample_size_int_group_follow_up	For trials, specify the sample size in the intervention group at the end of the follow-up time
	int_sample_size_comparison_group_follow_up	For trials, specify the sample size in the comparison group at the end of follow up time
Other	note_modeler	for modelers only, audience is modeler, not for correspondence
	note_sr	notes related to extraction, including assumptions, data adjustment, problems with source, any other notes that may be relevant, etc.

	extractor	uwnet id of person who extracted the data
Custom	custom_exp_meas_num	If the exposure level was assessed multiple times at a given time point (e.g., systolic blood pressure), specify the number of measurements at each time point.
	custom_exp_biomarker	If the exposure level was assessed via a biomarker, specify the full name of the biomarker.
	custom_exp_kilometer	Specify the geographical unit of measurement in kilometer (if applicable, e.g., satellite data).
	custom_exp_level_lower	if don't have a mean/midpoint exposure level can use this column in conjecture with the custom_exp_level_upper to enter in a range
	custom_exp_level_upper	if don't have a mean/midpoint exposure level can use this column in conjecture with the custom_exp_level_lower to enter in a range
	custom_unexp_level_lower	if don't have a mean/midpoint exposure level can use this column in conjecture with the custom_outcome_level_upper to enter in a range
	custom_unexp_level_upper	if don't have a mean/midpoint exposure level can use this column in conjecture with the custom_outcome_level_lower to enter in a range
	custom_prospective_lag	specify lag time between exposure assessment and outcome
	custom_age_demographer	A binary flag to identify if ages are presented in demographer notation or not in the source. This value is currently not used to adjust any age_start or age_end values, but in the future, that is the intention; 0 = article does not use demographer notation (4 = 4.00 not 4.99); 1 = article uses demographer notation (4=4.99 not 4.00)
	custom_bmi_menopause_free_text	free text field for bmi team
	custom_cvd_outcome	used for mapping cvd outcomes, free text field
	custom_dm_type	used for documenting diabeties type
	custom_dm_case_defn	used for documenting diabeties definitions, free text
	custom_pmid	to document Pubmed id
	custom_cvd_rep_high_risk	cvd specific, binary, if the study only includes people at high risk for CVD (1 for example if it is only among diabetics)
	custom_drug_class	class of drug being used in intervention, free text
	custom_outcome_primary	outcome is the primary outcome of RCT (1=yes, 0=no)
	custom_outcome_prespecified	outcome is the prespecified outcome of RCT (1=yes, 0=no)
	custom_multipollutant	Are any other pollutants controlled for in the model? 0=no, 1=yes
	custom_pollutants_controlled	if custom_multipollutant=1, list the pollutants controlled for
	custom_PM2.5_model_type	Describe the model used for exposure
	custom_assign_method	How do researchers assign participants to exp? (ex: by home address, by city, nearest zipcode centroid, etc.)
	custom_PM2.5_def	What metric are they using to measure PM2.5 (ex: mean of annual PM2.5 averages for 35-1 year prior to study)
	custom_lag	Do the authors take into account lag? If so, how?
	custom_PM2.5_min	All of these have to do with the spread of the PM2.5 exposure covered by the study. Minimum
	custom_PM2.5_5th	5th percentile
	custom_PM2.5_25th	25th percentile
	custom_PM2.5_50th	Median/50^{th}percentile
	custom_PM2.5_75th	75th percentile
	custom_PM2.5_95th	95th percentile
	custom_PM2.5_max	maximum
	custom_PM2.5_mean	Mean
	custom_PM2.5_stddev	Standard Deviation
	custom_PM2.5_other_measure	Any other measures of the distribution of PM2.5 amongst participants?
	custom_PM2.5_other_measure_description	If so, what are they? (ex: 10th, 90th, IQR)

Section 6: Study quality and risk of bias assessment

For each study that met the inclusion criteria, two reviewers assessed several indicators of bias during the extraction process. The full list of bias covariates assessed across all studies can be found in the extraction template (Supplementary Table 5).

To assist with a broad sense of data quality, we calculated a quality score for each study used in this analysis based on four study characteristics that were most applicable and likely to introduce bias (see supplementary Table 6). The overall score assessment was measured from 0 to 5, where 0 indicated the least bias and 5 indicated the most bias.

Supplementary Table 6. Study quality for every study used in the models

In this presentation of the data, there is one row per study/outcome pair

**Author**	**Year**	**Outcome**	**Exposure Measurement Score (multiple-0, single-1)**	**Exposure Assessment Score (objective-0 vs self-report-1)**	**Outcome Assessment Score (objective-0 vs self-report-1)**	**Confounders Score (age,sex,smoking,income,education-0; age,sex,smoking-1; age,sex-2)**	**Quality Score (best-0, worst-5)**
Barouti	2022	Diabetes mellitus	0	1	0	0	1
Barouti	2022	Diabetes mellitus	0	1	0	0	1
Bazzano	2008	Diabetes mellitus	0	1	0	2	3
Chen	2018	Diabetes mellitus	1	1	0	1	3
Cooper	2012	Diabetes mellitus	1	1	0	1	3
Hodge	2004	Diabetes mellitus	1	1	1	2	5
Kurotani	2013	Diabetes mellitus	0	1	1	2	4
Liu	2021	Diabetes mellitus	1	1	1	0	3
Liu	2004	Diabetes mellitus	1	1	1	2	5
Mamluk	2017	Diabetes mellitus	1	1	1	1	4
Meyer	2000	Diabetes mellitus	1	1	1	2	5
Montonen	2005	Diabetes mellitus	1	1	0	1	3
Olsson	2021	Diabetes mellitus	1	1	0	1	3
Qiao	2014	Diabetes mellitus	1	1	1	2	5
Villegas	2008	Diabetes mellitus	0	1	1	2	4
Ahmed	2020	Diabetes mellitus	0	1	1	1	3
Rayner	2020	Diabetes mellitus	0	1	1	2	4
Freedman	2007	Esophageal cancer	1	1	0	1	3

González	2006	Esophageal cancer	1	1	0	1	3
Sheikh	2019	Esophageal cancer	1	1	1	1	4
Steevens	2011	Esophageal cancer	1	1	0	1	3
Tran	2005	Esophageal cancer	1	1	0	2	4
Yamaji	2008	Esophageal cancer	1	1	0	2	4
Larsson	2009	Hemorrhagic stroke	1	1	0	2	4
Larsson	2013	Hemorrhagic stroke	1	1	0	1	3
Mizrahi	2009	Hemorrhagic stroke	1	1	0	1	3
Nagura	2009	Hemorrhagic stroke	1	1	0	1	3
Pham	2007	Hemorrhagic stroke	1	1	0	1	3
Yokoyama	2000	Hemorrhagic stroke	1	1	1	1	4
Zhang	2011	Hemorrhagic stroke	1	1	0	1	3
Bhupathiraju	2013	Ischemic heart disease	0	1	0	2	3
Dauchet	2010	Ischemic heart disease	1	1	0	2	4
Hansen	2010	Ischemic heart disease	1	1	0	1	3
Knekt	1994	Ischemic heart disease	1	1	0	2	4
Kobylecki	2015	Ischemic heart disease	1	1	0	1	3
Liu	2000	Ischemic heart disease	1	1	1	2	5
Miller	2017	Ischemic heart disease	1	1	0	1	3
Perez-Cornago	2021	Ischemic heart disease	1	1	0	0	2
Pietinen	1996	Ischemic heart disease	1	1	0	2	4
Rebello	2014	Ischemic heart disease	1	1	0	2	4
Sharma	2014	Ischemic heart disease	1	1	0	2	4
Sonestedt	2015	Ischemic heart disease	1	1	0	1	3
Stefler	2016	Ischemic heart disease	1	1	0	1	3
Tognon	2014	Ischemic heart disease	1	1	0	2	4
Yoshizaki	2020	Ischemic heart disease	1	1	0	1	3
Yu	2014	Ischemic heart disease	1	1	0	0	2
Zhang	2021	Ischemic heart disease	1	1	0	1	3
Johnsen	2003	Ischemic stroke	1	1	0	2	4

Joshipura	1999	Ischemic stroke	0	1	0	2	3
Larsson	2009	Ischemic stroke	1	1	0	2	4
Larsson	2013	Ischemic stroke	1	1	0	1	3
Mizrahi	2009	Ischemic stroke	1	1	0	1	3
Nagura	2009	Ischemic stroke	1	1	0	1	3
Pham	2007	Ischemic stroke	1	1	0	1	3
Steffen	2003	Ischemic stroke	1	1	0	1	3
Tong	2020	Ischemic stroke	1	1	0	0	2
Yokoyama	2000	Ischemic stroke	1	1	1	1	4
Yoshizaki	2020	Ischemic stroke	1	1	0	1	3
Zhang	2011	Ischemic stroke	1	1	0	1	3
Tong	2020	Hemorrhagic stroke	1	1	0	0	2
Yoshizaki	2020	Hemorrhagic stroke	1	1	0	1	3

Section 7: Results from individual studies

The health outcome, reference group exposure, alternative group exposure, log effect size, and log effect size SE identified from each report are provided in Supplementary Table 7.

Supplementary Table 7. Summary results from input studies

**Outcome**	**Alternative group exposure**	**Reference group exposure**	**Log effect size**	**Log effect size standard error**	**Study**
Diabetes mellitus	0 - 41 grams	42 - 78 grams	-0.29	0.15	Montonen 2005
Diabetes mellitus	0 - 41 grams	131 - 182 grams	-0.26	0.15	Montonen 2005
Diabetes mellitus	0 - 41 grams	79 - 130 grams	-0.07	0.14	Montonen 2005
Diabetes mellitus	0 - 146 grams	146 - 203 grams	0	0.05	Bazzano 2008
Diabetes mellitus	0 - 146 grams	203 - 257 grams	0.02	0.05	Bazzano 2008
Diabetes mellitus	0 - 146 grams	341 - 425 grams	0.05	0.05	Bazzano 2008
Diabetes mellitus	0 - 146 grams	257 - 341 grams	0.08	0.05	Bazzano 2008
Diabetes mellitus	0 - 152 grams	213 - 274 grams	-0.01	0.1	Meyer 2000
Diabetes mellitus	0 - 152 grams	153 - 212 grams	0.03	0.1	Meyer 2000
Diabetes mellitus	0 - 152 grams	368 - 460 grams	0.07	0.11	Meyer 2000
Diabetes mellitus	0 - 152 grams	275 - 367 grams	0.09	0.1	Meyer 2000
Diabetes mellitus	0 - 152 grams	226 - 306 grams	-0.02	0.09	Liu 2004
Diabetes mellitus	0 - 152 grams	306 - 438 grams	-0.01	0.09	Liu 2004

Diabetes mellitus	0 - 152 grams	152 - 226 grams	0.01	0.08	Liu 2004
Diabetes mellitus	0 - 152 grams	438 - 569 grams	0.03	0.09	Liu 2004
Diabetes mellitus	0 - 212 grams	300 - 387 grams	-0.39	0.08	Villegas 2008
Diabetes mellitus	0 - 212 grams	387 - 517 grams	-0.33	0.08	Villegas 2008
Diabetes mellitus	0 - 212 grams	517 - 646 grams	-0.33	0.08	Villegas 2008
Diabetes mellitus	0 - 212 grams	212 - 300 grams	-0.3	0.08	Villegas 2008
Diabetes mellitus	0 - 138 grams	209 - 312 grams	-0.27	0.18	Kurotani 2013
Diabetes mellitus	0 - 110 grams	267 - 358 grams	-0.21	0.17	Kurotani 2013
Diabetes mellitus	0 - 110 grams	175 - 267 grams	-0.08	0.14	Kurotani 2013
Diabetes mellitus	0 - 110 grams	110 - 175 grams	-0.07	0.12	Kurotani 2013
Diabetes mellitus	0 - 138 grams	312 - 415 grams	-0.01	0.2	Kurotani 2013
Diabetes mellitus	0 - 138 grams	138 - 209 grams	0.04	0.15	Kurotani 2013
Diabetes mellitus	0 - 222 grams	536 - 682 grams	-0.13	0.19	Hodge 2004
Diabetes mellitus	0 - 222 grams	383 - 529 grams	-0.03	0.18	Hodge 2004
Diabetes mellitus	0 - 222 grams	230 - 375 grams	0.09	0.17	Hodge 2004
Diabetes mellitus	0 - 230 grams	231 - 460 grams	0.1	0.07	Qiao 2014
Diabetes mellitus	0 - 100 grams	100 - 155 grams	-0.08	0.05	Cooper 2012
Diabetes mellitus	0 - 100 grams	155 - 238 grams	-0.07	0.06	Cooper 2012
Diabetes mellitus	0 - 100 grams	238 - 320 grams	-0.06	0.06	Cooper 2012
Diabetes mellitus	0 - 112 grams	200 - 312 grams	-0.13	0.03	Mamluk 2017
Diabetes mellitus	0 - 112 grams	120 - 192 grams	-0.08	0.03	Mamluk 2017
Diabetes mellitus	0 - 112 grams	320 - 432 grams	-0.08	0.03	Mamluk 2017
Diabetes mellitus	0 - 112 grams	120 - 192 grams	-0.01	0.12	Mamluk 2017
Diabetes mellitus	0 - 112 grams	320 - 432 grams	0.05	0.14	Mamluk 2017
Diabetes mellitus	0 - 112 grams	200 - 312 grams	0.1	0.13	Mamluk 2017
Diabetes mellitus	0 - 102 grams	102 - 142 grams	-0.08	0.07	Olsson 2021
Diabetes mellitus	0 - 102 grams	142 - 184 grams	-0.12	0.07	Olsson 2021
Diabetes mellitus	0 - 102 grams	184 - 246 grams	-0.06	0.07	Olsson 2021
Diabetes mellitus	0 - 102 grams	246 - 1177 grams	-0.16	0.08	Olsson 2021
Diabetes mellitus	0 - 102 grams	102 - 142 grams	-0.05	0.07	Olsson 2021
Diabetes mellitus	0 - 102 grams	142 - 184 grams	-0.11	0.07	Olsson 2021
Diabetes mellitus	0 - 102 grams	184 - 246 grams	0.02	0.07	Olsson 2021
Diabetes mellitus	0 - 102 grams	246 - 1177 grams	0.05	0.07	Olsson 2021
Diabetes mellitus	0 - 167 grams	167 - 205 grams	-0.37	0.16	Liu 2021
Diabetes mellitus	0 - 71 grams	71 - 138 grams	-0.21	0.11	Barouti 2022
Diabetes mellitus	0 - 71 grams	138 - 216 grams	-0.19	0.11	Barouti 2022
Diabetes mellitus	0 - 100 grams	100 - 181 grams	0.02	0.12	Barouti 2022
Diabetes mellitus	0 - 100 grams	181 - 279 grams	0.13	0.12	Barouti 2022
Diabetes mellitus	0 - 90 grams	90 - 120 grams	0.15	0.04	Chen 2018
Diabetes mellitus	0 - 90 grams	120 - 152 grams	-0.02	0.05	Chen 2018
Diabetes mellitus	0 - 90 grams	152 - 202 grams	0.02	0.05	Chen 2018

Diabetes mellitus	0 - 90 grams	202 - 268 grams	0.08	0.05	Chen 2018
Diabetes mellitus	0 - 154 grams	154 - 308 grams	0.48	0.24	Ahmed 2020
Diabetes mellitus	0 - 154 grams	154 - 308 grams	0.17	0.19	Ahmed 2020
Diabetes mellitus	3 - 3 grams	1 - 1 grams	0.03	0.1	Rayner 2020
Diabetes mellitus	3 - 3 grams	2 - 2 grams	0.02	0.11	Rayner 2020
Diabetes mellitus	3 - 3 grams	2 - 2 grams	0.01	0.08	Rayner 2020
Ischemic heart disease	0 - 77 grams	78 - 137 grams	-0.87	0.36	Knekt 1994
Ischemic heart disease	0 - 61 grams	117 - 172 grams	-0.42	0.19	Knekt 1994
Ischemic heart disease	0 - 77 grams	137 - 196 grams	-0.42	0.32	Knekt 1994
Ischemic heart disease	0 - 61 grams	62 - 117 grams	-0.17	0.17	Knekt 1994
Ischemic heart disease	0 - 38 grams	38 - 77 grams	-0.12	0.07	Kobylecki 2015
Ischemic heart disease	0 - 38 grams	77 - 115 grams	-0.19	0.07	Kobylecki 2015
Ischemic heart disease	0 - 38 grams	153 - 230 grams	-0.13	0.08	Kobylecki 2015
Ischemic heart disease	0 - 143 grams	197 - 252 grams	-0.24	0.06	Bhupathiraju 2013
Ischemic heart disease	0 - 143 grams	252 - 338 grams	-0.17	0.07	Bhupathiraju 2013
Ischemic heart disease	0 - 143 grams	338 - 424 grams	-0.16	0.07	Bhupathiraju 2013
Ischemic heart disease	0 - 143 grams	143 - 197 grams	-0.12	0.06	Bhupathiraju 2013
Ischemic heart disease	0 - 134 grams	340 - 432 grams	-0.08	0.06	Bhupathiraju 2013
Ischemic heart disease	0 - 134 grams	189 - 247 grams	-0.04	0.05	Bhupathiraju 2013
Ischemic heart disease	0 - 134 grams	247 - 340 grams	-0.04	0.06	Bhupathiraju 2013
Ischemic heart disease	0 - 134 grams	134 - 189 grams	-0.01	0.05	Bhupathiraju 2013
Ischemic heart disease	0 - 37 grams	127 - 169 grams	-0.51	0.14	Pietinen 1996
Ischemic heart disease	0 - 37 grams	59 - 85 grams	-0.37	0.13	Pietinen 1996
Ischemic heart disease	0 - 37 grams	85 - 127 grams	-0.26	0.13	Pietinen 1996
Ischemic heart disease	0 - 37 grams	37 - 59 grams	0.03	0.11	Pietinen 1996
Ischemic heart disease	0 - 176 grams	506 - 659 grams	-0.31	0.12	Sharma 2014
Ischemic heart disease	0 - 176 grams	261 - 352 grams	-0.27	0.1	Sharma 2014
Ischemic heart disease	0 - 176 grams	261 - 352 grams	-0.27	0.12	Sharma 2014
Ischemic heart disease	0 - 176 grams	352 - 506 grams	-0.26	0.13	Sharma 2014
Ischemic heart disease	0 - 176 grams	352 - 506 grams	-0.2	0.1	Sharma 2014
Ischemic heart disease	0 - 176 grams	176 - 261 grams	-0.07	0.09	Sharma 2014
Ischemic heart disease	0 - 176 grams	506 - 659 grams	-0.05	0.14	Sharma 2014
Ischemic heart disease	0 - 176 grams	176 - 261 grams	-0.01	0.11	Sharma 2014
Ischemic heart disease	0 - 153 grams	226 - 306 grams	-0.6	0.33	Liu 2000
Ischemic heart disease	0 - 153 grams	306 - 441 grams	-0.14	0.29	Liu 2000
Ischemic heart disease	0 - 153 grams	441 - 575 grams	-0.13	0.29	Liu 2000
Ischemic heart disease	0 - 153 grams	153 - 226 grams	-0.06	0.28	Liu 2000
Ischemic heart disease	0 - 61 grams	77 - 99 grams	-0.43	0.2	Dauchet 2010
Ischemic heart disease	0 - 61 grams	77 - 99 grams	-0.34	0.29	Dauchet 2010
Ischemic heart disease	0 - 61 grams	115 - 137 grams	-0.33	0.24	Dauchet 2010
Ischemic heart disease	0 - 61 grams	77 - 99 grams	-0.05	0.22	Dauchet 2010

Ischemic heart disease	0 - 61 grams	115 - 137 grams	0.22	0.27	Dauchet 2010
Ischemic heart disease	0 - 61 grams	115 - 137 grams	0.25	0.21	Dauchet 2010
Ischemic heart disease	80 - 162 grams	183 - 318 grams	-0.2	0.2	Stefler 2016
Ischemic heart disease	80 - 162 grams	138 - 234 grams	-0.06	0.18	Stefler 2016
Ischemic heart disease	80 - 162 grams	263 - 495 grams	0	0.21	Stefler 2016
Ischemic heart disease	0 - 48 grams	99 - 797 grams	-0.37	0.15	Rebello 2014
Ischemic heart disease	0 - 48 grams	62 - 77 grams	-0.22	0.1	Rebello 2014
Ischemic heart disease	0 - 48 grams	99 - 668 grams	-0.17	0.11	Rebello 2014
Ischemic heart disease	0 - 48 grams	62 - 77 grams	-0.17	0.13	Rebello 2014
Ischemic heart disease	0 - 48 grams	77 - 99 grams	-0.15	0.13	Rebello 2014
Ischemic heart disease	0 - 48 grams	77 - 99 grams	-0.14	0.1	Rebello 2014
Ischemic heart disease	0 - 48 grams	48 - 62 grams	-0.08	0.09	Rebello 2014
Ischemic heart disease	0 - 48 grams	48 - 62 grams	-0.07	0.13	Rebello 2014
Ischemic heart disease	0 - 192 grams	192 - 384 grams	-0.31	0.16	Tognon 2014
Ischemic heart disease	0 - 175 grams	175 - 252 grams	0.14	0.22	Yu 2014
Ischemic heart disease	0 - 175 grams	252 - 360 grams	-0.26	0.24	Yu 2014
Ischemic heart disease	0 - 175 grams	360 - 498 grams	-0.19	0.24	Yu 2014
Ischemic heart disease	0 - 206 grams	206 - 298 grams	-0.05	0.19	Yu 2014
Ischemic heart disease	0 - 206 grams	298 - 423 grams	-0.39	0.21	Yu 2014
Ischemic heart disease	0 - 206 grams	423 - 581 grams	0.02	0.19	Yu 2014
Ischemic heart disease	0 - 77 grams	77 - 154 grams	0.06	0.07	Miller 2017
Ischemic heart disease	0 - 77 grams	154 - 231 grams	0.12	0.07	Miller 2017
Ischemic heart disease	0 - 77 grams	231 - 308 grams	-0.07	0.09	Miller 2017
Ischemic heart disease	0 - 98 grams	98 - 144 grams	-0.05	0.07	Sonestedt 2015
Ischemic heart disease	0 - 98 grams	144 - 188 grams	-0.01	0.07	Sonestedt 2015
Ischemic heart disease	0 - 98 grams	188 - 272 grams	-0.02	0.07	Sonestedt 2015
Ischemic heart disease	0 - 98 grams	272 - 392 grams	-0.01	0.08	Sonestedt 2015
Ischemic heart disease	0 - 143 grams	143 - 254 grams	0.17	0.21	Yoshizaki 2020
Ischemic heart disease	0 - 143 grams	254 - 386 grams	0.04	0.24	Yoshizaki 2020
Ischemic heart disease	0 - 169 grams	169 - 285 grams	0.22	0.38	Yoshizaki 2020
Ischemic heart disease	0 - 169 grams	285 - 422 grams	0.17	0.41	Yoshizaki 2020
Ischemic heart disease	0 - 154 grams	154 - 231 grams	-0.04	0.02	Zhang 2021
Ischemic heart disease	0 - 154 grams	231 - 308 grams	-0.01	0.02	Zhang 2021
Ischemic heart disease	0 - 154 grams	308 - 385 grams	-0.05	0.02	Zhang 2021
Ischemic heart disease	0 - 116 grams	116 - 192 grams	0.01	0.03	Perez-Cornago 2021
Ischemic heart disease	0 - 116 grams	192 - 308 grams	-0.07	0.04	Perez-Cornago 2021
Ischemic heart disease	0 - 116 grams	308 - 424 grams	-0.04	0.05	Perez-Cornago 2021
Ischemic heart disease	0 - 96 grams	96 - 148 grams	0.06	0.1	Hansen 2010
Ischemic heart disease	0 - 96 grams	148 - 211 grams	0.03	0.1	Hansen 2010
Ischemic heart disease	0 - 96 grams	211 - 274 grams	-0.07	0.11	Hansen 2010
Ischemic heart disease	0 - 110 grams	110 - 167 grams	0.14	0.16	Hansen 2010

Ischemic heart disease	0 - 110 grams	167 - 237 grams	-0.13	0.19	Hansen 2010
Ischemic heart disease	0 - 110 grams	237 - 307 grams	0.09	0.2	Hansen 2010
Ischemic stroke	0 - 19 grams	19 - 31 grams	-0.14	0.16	Nagura 2009
Ischemic stroke	0 - 19 grams	45 - 66 grams	0.03	0.17	Nagura 2009
Ischemic stroke	0 - 19 grams	31 - 45 grams	0.22	0.16	Nagura 2009
Ischemic stroke	0 - 44 grams	45 - 84 grams	-0.36	0.15	Mizrahi 2009
Ischemic stroke	1 - 56 grams	57 - 95 grams	-0.36	0.15	Mizrahi 2009
Ischemic stroke	0 - 44 grams	85 - 137 grams	-0.25	0.15	Mizrahi 2009
Ischemic stroke	0 - 44 grams	138 - 535 grams	-0.08	0.16	Mizrahi 2009
Ischemic stroke	0 - 11 grams	33 - 66 grams	-0.17	0.09	Zhang 2011
Ischemic stroke	0 - 11 grams	77 - 109 grams	-0.17	0.11	Zhang 2011
Ischemic stroke	0 - 11 grams	11 - 22 grams	-0.02	0.09	Zhang 2011
Ischemic stroke	0 - 157 grams	310 - 421 grams	-0.27	0.18	Joshipura 1999
Ischemic stroke	0 - 134 grams	188 - 264 grams	-0.27	0.23	Joshipura 1999
Ischemic stroke	0 - 134 grams	264 - 364 grams	-0.21	0.23	Joshipura 1999
Ischemic stroke	0 - 157 grams	222 - 310 grams	-0.13	0.17	Joshipura 1999
Ischemic stroke	0 - 157 grams	421 - 533 grams	-0.12	0.18	Joshipura 1999
Ischemic stroke	0 - 134 grams	364 - 464 grams	-0.11	0.23	Joshipura 1999
Ischemic stroke	0 - 134 grams	134 - 188 grams	-0.01	0.22	Joshipura 1999
Ischemic stroke	0 - 157 grams	157 - 222 grams	0.21	0.15	Joshipura 1999
Ischemic stroke	0 - 92 grams	264 - 338 grams	-0.16	0.2	Johnsen 2003
Ischemic stroke	0 - 92 grams	92 - 140 grams	-0.09	0.18	Johnsen 2003
Ischemic stroke	0 - 92 grams	140 - 188 grams	-0.07	0.18	Johnsen 2003
Ischemic stroke	0 - 92 grams	188 - 264 grams	-0.03	0.19	Johnsen 2003
Ischemic stroke	0 - 37 grams	126 - 168 grams	-0.29	0.06	Larsson 2009
Ischemic stroke	0 - 37 grams	59 - 84 grams	-0.11	0.06	Larsson 2009
Ischemic stroke	0 - 37 grams	84 - 126 grams	-0.09	0.06	Larsson 2009
Ischemic stroke	0 - 37 grams	37 - 59 grams	-0.06	0.06	Larsson 2009
Ischemic stroke	0 - 153 grams	479 - 632 grams	-0.06	0.28	Steffen 2003
Ischemic stroke	0 - 153 grams	326 - 479 grams	0.04	0.25	Steffen 2003
Ischemic stroke	0 - 153 grams	230 - 326 grams	0.1	0.24	Steffen 2003
Ischemic stroke	0 - 153 grams	153 - 230 grams	0.44	0.22	Steffen 2003
Ischemic stroke	0 - 103 grams	165 - 226 grams	-0.13	0.06	Larsson 2013
Ischemic stroke	0 - 103 grams	326 - 425 grams	-0.13	0.07	Larsson 2013
Ischemic stroke	0 - 103 grams	226 - 326 grams	-0.06	0.06	Larsson 2013
Ischemic stroke	0 - 103 grams	103 - 165 grams	-0.05	0.06	Larsson 2013
Ischemic stroke	0 - 77 grams	77 - 115 grams	0.18	0.2	Pham 2007
Ischemic stroke	0 - 22 grams	66 - 77 grams	-0.71	0.61	Yokoyama 2000
Ischemic stroke	0 - 22 grams	66 - 77 grams	-0.19	1.03	Yokoyama 2000
Ischemic stroke	0 - 22 grams	33 - 55 grams	-0.16	1.27	Yokoyama 2000
Ischemic stroke	0 - 22 grams	22 - 55 grams	-0.04	0.75	Yokoyama 2000

Ischemic stroke	0 - 92 grams	92 - 136 grams	-0.06	0.05	Tong 2020
Ischemic stroke	0 - 92 grams	136 - 192 grams	-0.09	0.05	Tong 2020
Ischemic stroke	0 - 92 grams	192 - 286 grams	-0.13	0.05	Tong 2020
Ischemic stroke	0 - 92 grams	286 - 380 grams	-0.14	0.06	Tong 2020
Ischemic stroke	0 - 143 grams	143 - 254 grams	-0.09	0.15	Yoshizaki 2020
Ischemic stroke	0 - 143 grams	254 - 386 grams	0.01	0.16	Yoshizaki 2020
Ischemic stroke	0 - 169 grams	169 - 285 grams	-0.2	0.19	Yoshizaki 2020
Ischemic stroke	0 - 169 grams	285 - 422 grams	-0.06	0.2	Yoshizaki 2020
Hemorrhagic stroke	0 - 19 grams	31 - 47 grams	-0.13	0.16	Nagura 2009
Hemorrhagic stroke	0 - 19 grams	19 - 31 grams	0.09	0.15	Nagura 2009
Hemorrhagic stroke	0 - 19 grams	47 - 63 grams	0.2	0.16	Nagura 2009
Hemorrhagic stroke	0 - 44 grams	45 - 84 grams	-0.04	0.39	Mizrahi 2009
Hemorrhagic stroke	1 - 56 grams	57 - 95 grams	-0.04	0.39	Mizrahi 2009
Hemorrhagic stroke	0 - 44 grams	85 - 137 grams	0.37	0.36	Mizrahi 2009
Hemorrhagic stroke	0 - 44 grams	138 - 535 grams	0.37	0.38	Mizrahi 2009
Hemorrhagic stroke	0 - 11 grams	77 - 109 grams	-0.34	0.22	Zhang 2011
Hemorrhagic stroke	0 - 11 grams	33 - 66 grams	-0.22	0.19	Zhang 2011
Hemorrhagic stroke	0 - 11 grams	11 - 22 grams	-0.15	0.17	Zhang 2011
Hemorrhagic stroke	0 - 37 grams	126 - 168 grams	-0.48	0.23	Larsson 2009
Hemorrhagic stroke	0 - 37 grams	84 - 126 grams	-0.31	0.17	Larsson 2009
Hemorrhagic stroke	0 - 37 grams	37 - 59 grams	-0.09	0.15	Larsson 2009
Hemorrhagic stroke	0 - 37 grams	59 - 84 grams	-0.03	0.15	Larsson 2009
Hemorrhagic stroke	0 - 103 grams	326 - 425 grams	-0.13	0.18	Larsson 2013
Hemorrhagic stroke	0 - 103 grams	103 - 165 grams	-0.05	0.14	Larsson 2013
Hemorrhagic stroke	0 - 103 grams	165 - 226 grams	-0.04	0.16	Larsson 2013
Hemorrhagic stroke	0 - 103 grams	226 - 326 grams	-0.02	0.17	Larsson 2013
Hemorrhagic stroke	0 - 77 grams	77 - 77 grams	0.1	0.31	Pham 2007
Hemorrhagic stroke	0 - 22 grams	66 - 77 grams	-0.62	1.04	Yokoyama 2000
Hemorrhagic stroke	0 - 22 grams	66 - 77 grams	-0.4	1.09	Yokoyama 2000
Hemorrhagic stroke	0 - 22 grams	33 - 55 grams	0.23	1.29	Yokoyama 2000
Hemorrhagic stroke	0 - 92 grams	92 - 136 grams	-0.19	0.09	Tong 2020
Hemorrhagic stroke	0 - 92 grams	136 - 192 grams	0.03	0.08	Tong 2020
Hemorrhagic stroke	0 - 92 grams	192 - 286 grams	-0.15	0.09	Tong 2020
Hemorrhagic stroke	0 - 92 grams	286 - 380 grams	0	0.1	Tong 2020
Hemorrhagic stroke	0 - 143 grams	143 - 254 grams	0.39	0.21	Yoshizaki 2020
Hemorrhagic stroke	0 - 143 grams	254 - 386 grams	0.58	0.23	Yoshizaki 2020
Hemorrhagic stroke	0 - 169 grams	169 - 285 grams	-0.2	0.23	Yoshizaki 2020
Hemorrhagic stroke	0 - 169 grams	285 - 422 grams	0.12	0.24	Yoshizaki 2020
Esophageal cancer	0 - 125 grams	164 - 202 grams	-0.58	0.36	Steevens 2011

Esophageal cancer	0 - 125 grams	260 - 318 grams	-0.53	0.3	Steevens 2011
Esophageal cancer	0 - 125 grams	164 - 202 grams	-0.49	0.28	Steevens 2011
Esophageal cancer	0 - 125 grams	260 - 318 grams	-0.49	0.39	Steevens 2011
Esophageal cancer	0 - 125 grams	125 - 164 grams	-0.34	0.27	Steevens 2011
Esophageal cancer	0 - 125 grams	125 - 164 grams	-0.3	0.34	Steevens 2011
Esophageal cancer	0 - 125 grams	202 - 260 grams	-0.19	0.26	Steevens 2011
Esophageal cancer	0 - 125 grams	202 - 260 grams	0.31	0.3	Steevens 2011
Esophageal cancer	0 - 139 grams	321 - 381 grams	-0.56	0.37	Freedman 2007
Esophageal cancer	0 - 139 grams	236 - 321 grams	-0.45	0.33	Freedman 2007
Esophageal cancer	0 - 139 grams	186 - 236 grams	-0.36	0.3	Freedman 2007
Esophageal cancer	0 - 139 grams	139 - 186 grams	-0.25	0.27	Freedman 2007
Esophageal cancer	0 - 139 grams	321 - 381 grams	-0.08	0.25	Freedman 2007
Esophageal cancer	0 - 139 grams	236 - 321 grams	-0.04	0.22	Freedman 2007
Esophageal cancer	0 - 139 grams	186 - 236 grams	0.08	0.21	Freedman 2007
Esophageal cancer	0 - 139 grams	139 - 186 grams	0.16	0.2	Freedman 2007
Esophageal cancer	0 - 126 grams	226 - 324 grams	-0.39	0.25	Yamaji 2008
Esophageal cancer	0 - 126 grams	126 - 226 grams	-0.22	0.22	Yamaji 2008
Esophageal cancer	0 - 115 grams	115 - 154 grams	-0.07	0.06	Tran 2005
Esophageal cancer	0 - 115 grams	154 - 192 grams	0.01	0.06	Tran 2005
Esophageal cancer	0 - 115 grams	192 - 231 grams	0.02	0.08	Tran 2005
Esophageal cancer	240 - 320 grams	80 - 160 grams	0.46	0.12	Sheikh 2019
Esophageal cancer	240 - 320 grams	40 - 80 grams	0.48	0.23	Sheikh 2019
Esophageal cancer	0 - 136 grams	136 - 243 grams	-0.13	0.31	González 2006

Section 8: Sensitivity results

Supplementary Figure 1: Vegetable consumption and ischemic stroke: Non-trimmed data.

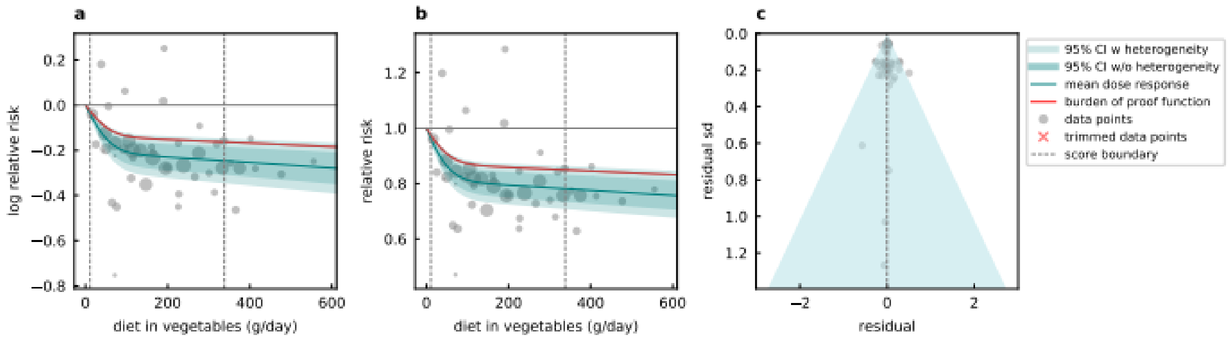

a, log-relative risk function
b, relative risk function
c, A modified funnel plot showing the residuals (relative to 0) on the x-axis and the estimated standard deviation (SD) that includes reported SD and between-study heterogeneity on the y-axis

Supplementary Figure 2: Vegetable consumption and ischemic heart disease: Non-trimmed data.

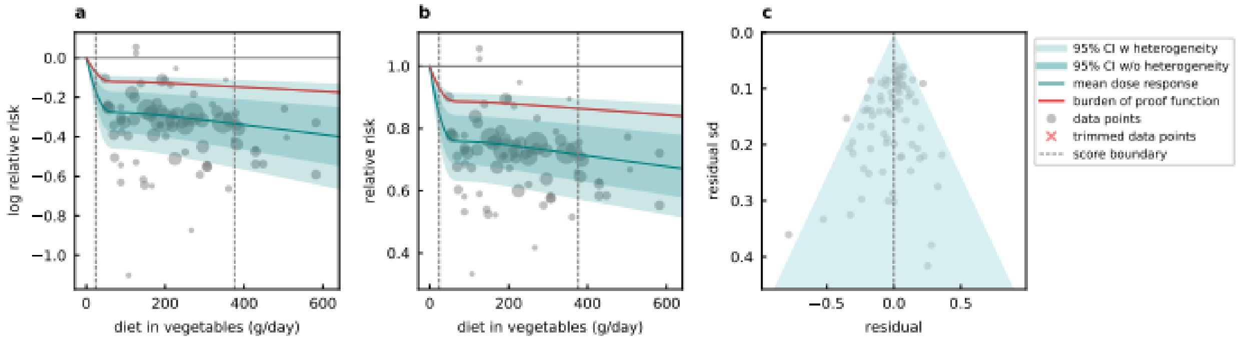

a, log-relative risk function
b, relative risk function
c, A modified funnel plot showing the residuals (relative to 0) on the x-axis and the estimated standard deviation (SD) that includes reported SD and between-study heterogeneity on the y-axis

Supplementary Figure 3: Vegetable consumption and hemorrhagic stroke: Non-trimmed data.

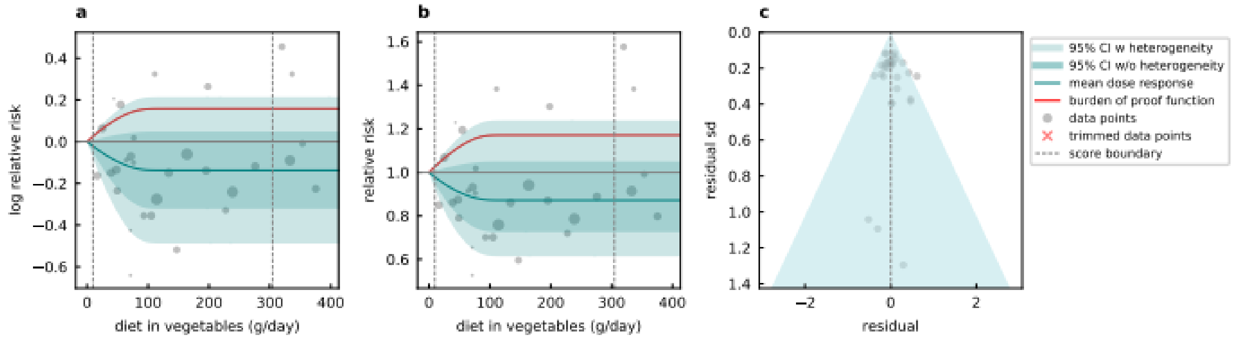

a, log-relative risk function
b, relative risk function
c, A modified funnel plot showing the residuals (relative to 0) on the x-axis and the estimated standard deviation (SD) that includes reported SD and between-study heterogeneity on the y-axis

Supplementary Figure 4: Vegetable consumption and esophageal cancer: Non-trimmed data.

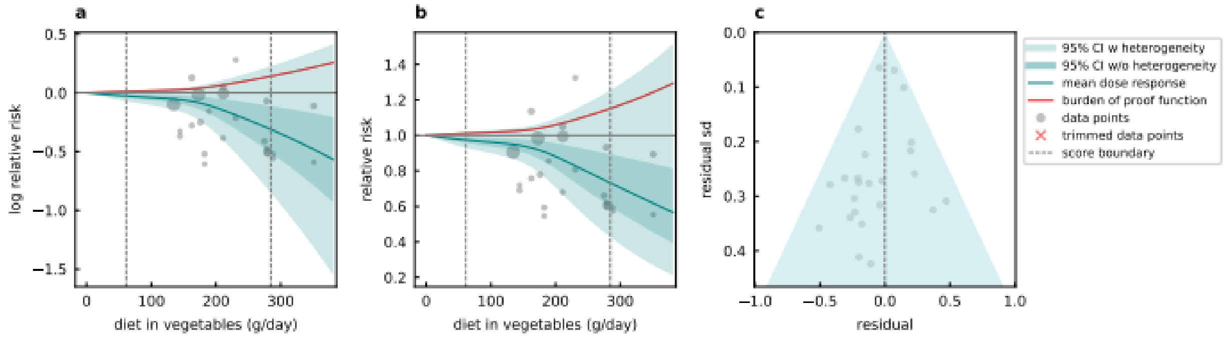

a, log-relative risk function
b, relative risk function
c, A modified funnel plot showing the residuals (relative to 0) on the x-axis and the estimated standard deviation (SD) that includes reported SD and between-study heterogeneity on the y-axis

Supplementary Figure 5: Vegetable consumption and type 2 diabetes: Non-trimmed data.

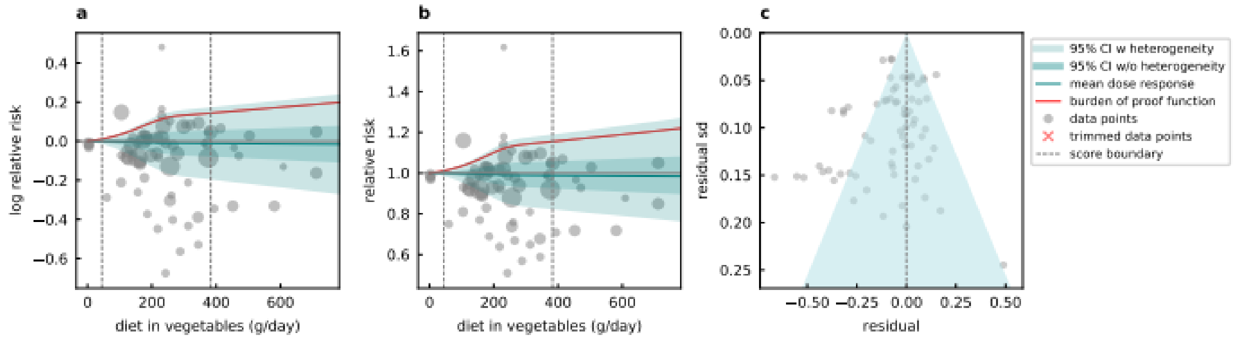

a, log-relative risk function
b, relative risk function
c, A modified funnel plot showing the residuals (relative to 0) on the x-axis and the estimated standard deviation (SD) that includes reported SD and between-study heterogeneity on the y-axis

Supplementary Figure 6: caloric adjusted vs unadjusted

a **b**

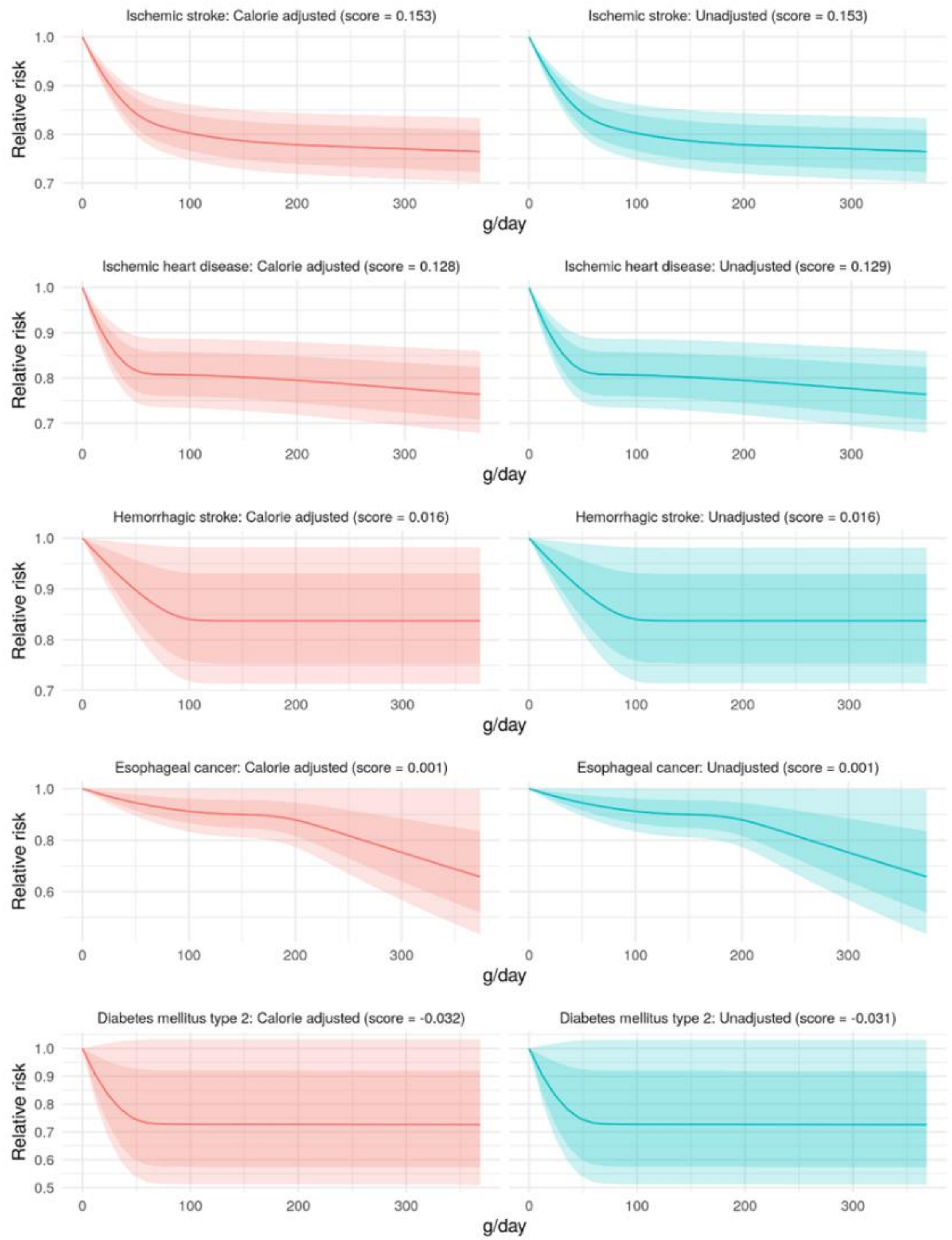

Sensitivity analysis results comparing calorie adjusted (pink) and calorie intake unadjusted (Turquoise) models for vegetables and five health outcomes (ischemic stroke, ischemic heart diseases, hemorrhagic stroke and esophageal cancer, diabetes mellitus type 2).
