# Supplementary material for: Health effects associated with vegetable consumption: a Burden of Proof study
Source: Nat Med. 2022 Oct 10;28(10):2066–74. doi: 10.1038/s41591-022-01970-5 (PMC9556321; doi:10.1038/s41591-022-01970-5)
Supplement: Supplementary file 2 — Reporting Summary [file 41591_2022_1970_MOESM2_ESM.pdf]

Corresponding author(s): Jeffrey D. Stanaway
Last updated by author(s): Jul 22, 2022

Reporting Summary

Nature Portfolio wishes to improve the reproducibility of the work that we publish. This form provides structure for consistency and transparency in reporting. For further information on Nature Portfolio policies, see our Editorial Policies and the Editorial Policy Checklist.

Statistics

For all statistical analyses, confirm that the following items are present in the figure legend, table legend, main text, or Methods section.

n/a	Confirmed	
☐	☒	The exact sample size (*n*) for each experimental group/condition, given as a discrete number and unit of measurement
☐	☒	A statement on whether measurements were taken from distinct samples or whether the same sample was measured repeatedly
☐	☒	The statistical test(s) used AND whether they are one- or two-sided<br>*Only common tests should be described solely by name; describe more complex techniques in the Methods section.*
☐	☒	A description of all covariates tested
☐	☒	A description of any assumptions or corrections, such as tests of normality and adjustment for multiple comparisons
☐	☒	A full description of the statistical parameters including central tendency (e.g. means) or other basic estimates (e.g. regression coefficient) AND variation (e.g. standard deviation) or associated estimates of uncertainty (e.g. confidence intervals)
☐	☒	For null hypothesis testing, the test statistic (e.g. *F*, *t*, *r*) with confidence intervals, effect sizes, degrees of freedom and *P* value noted<br>*Give P values as exact values whenever suitable.*
☐	☒	For Bayesian analysis, information on the choice of priors and Markov chain Monte Carlo settings
☒	☐	For hierarchical and complex designs, identification of the appropriate level for tests and full reporting of outcomes
☐	☒	Estimates of effect sizes (e.g. Cohen's *d*, Pearson's *r*), indicating how they were calculated

Our web collection on statistics for biologists contains articles on many of the points above.

Software and code

Policy information about availability of computer code

Data collection	No primary data collection was carried out for this analysis.
Data analysis	All code used for these analyses is publicly available online (https://github.com/ihmeuw-msca/burden-of-proof). This includes code for the meta-regression engine, the model specification interface, both parts of the data processing, and risk-specific custom code, as appropriate. Analyses were carried out using R version 3.6.1, Python version 3.8, and Stata version 17.<br><br>To validate key aspects of the meta-regression model used in this analysis, the following packages were used, as described in Zheng et al: metafor (R package available for download at https://www.jstatsoft.org/article/view/v036i03) and dosmesreta (R package available for download at https://www.jstatsoft.org/article/view/v072c01).

For manuscripts utilizing custom algorithms or software that are central to the research but not yet described in published literature, software must be made available to editors and reviewers. We strongly encourage code deposition in a community repository (e.g. GitHub). See the Nature Portfolio guidelines for submitting code & software for further information.

Data

Policy information about availability of data

All manuscripts must include a data availability statement. This statement should provide the following information, where applicable:

- Accession codes, unique identifiers, or web links for publicly available datasets
- A description of any restrictions on data availability
- For clinical datasets or third party data, please ensure that the statement adheres to our policy

The findings from this study were produced using data available in public online repositories and in the published literature. Study sources and citations for each risk-outcome pair can be downloaded using the “download” button on each risk curve page at https://vizhub.healthdata.org/burden-of-proof/. Study characteristics for all input data used in the analyses are also provided in Supplementary Information Table S5. See Supplementary Information Table S4 for a template of the data collection form.

Field-specific reporting

Please select the one below that is the best fit for your research. If you are not sure, read the appropriate sections before making your selection.

☒ Life sciences ☐ Behavioural & social sciences ☐ Ecological, evolutionary & environmental sciences

For a reference copy of the document with all sections, see nature.com/documents/nr-reporting-summary-flat.pdf

Life sciences study design

All studies must disclose on these points even when the disclosure is negative.

Sample size

No sample size calculation was performed for this meta-analysis; all available datasets meeting inclusion criteria or included. As reported in the results section, the number of data points were as follows:
Vegetables and IHD: 1,952,226 participants
Vegetables and ischemic stroke: 829,610 participants
Vegetables and type 2 diabetes: 1,025,899 participants
Vegetables and hemorrhagic stroke: 646,338 participants
Vegetables and esophageal cancer: 818,059 participants

Data exclusions

Studies were eligible for inclusion if they used suitable exposure and outcome definitions, provided some measure of uncertainty (e.g. sample size, standard error, or confidence intervals), and reported a relative risk (or odds ratio) for which the exposed and unexposed groups were defined. Where multiple studies provided relative risk estimates from the same cohort, we included only the study that captured the largest sample or follow-up time so as not to include duplicate data.

As identified in Supplementary Information Figures S3-S6, studies were excluded based on: no outcome of exposure, no outcome of interest, wrong exposure definition, no study design of interest, unmeasurable, duplicate cohorts.

Replication

This is a meta-analysis of existing studies with many years of cohort and other data. When re-applying the method to the same data, we get the same results.

Randomization

This analysis is a meta-analysis of existing studies and thus, there were no experimental groups.

Blinding

N/A. Blinding was not relevant to this study, as we did not collect primary data.

Reporting for specific materials, systems and methods

We require information from authors about some types of materials, experimental systems and methods used in many studies. Here, indicate whether each material, system or method listed is relevant to your study. If you are not sure if a list item applies to your research, read the appropriate section before selecting a response.

Materials & experimental systems

n/a	Involved in the study	
☒	☐	Antibodies
☒	☐	Eukaryotic cell lines
☒	☐	Palaeontology and archaeology
☒	☐	Animals and other organisms
☒	☐	Human research participants
☒	☐	Clinical data
☒	☐	Dual use research of concern

Methods

n/a	Involved in the study	
☒	☐	ChIP-seq
☒	☐	Flow cytometry
☒	☐	MRI-based neuroimaging
